# Alcohol-attributable cancer risk and burden estimates for Australia’s updated alcohol consumption guidelines

**DOI:** 10.1038/s41416-026-03403-3

**Published:** 2026-04-10

**Authors:** Peter Sarich, Karen Canfell, Sam Egger, Emily Banks, Grace Joshy, Lyndal Wellard-Cole, Clare Hughes, Nehmat Houssami, Paul Grogan, Marianne F. Weber

**Affiliations:** 1 Cancer Elimination Collaboration, Sydney School of Public Health, Faculty of Medicine and Health, The University of Sydney, Newtown, NSW Australia; 2 The Daffodil Centre, The University of Sydney, A Joint Venture with Cancer Council NSW, Sydney, NSW Australia; 3 National Centre for Epidemiology and Population Health, Australian National University, Canberra, ACT Australia; 4 Cancer Prevention and Advocacy Division, Cancer Council NSW, Sydney, NSW Australia; 5 Discipline of Nutrition and Dietetics, Susan Wakil School of Nursing and Midwifery, Faculty of Medicine and Health, The University of Sydney, Sydney, NSW Australia; 6 Sydney School of Public Health, Faculty of Medicine and Health, The University of Sydney, Sydney, NSW Australia

**Keywords:** Risk factors, Cancer, Epidemiology

## Abstract

**Background:**

The Australian alcohol health guidelines were revised in 2020 to recommend a maximum of 10 drinks/week. We calculated estimates of cancer caused by alcohol use in Australia for the updated recommended limits.

**Methods:**

Cox regression models were used to estimate hazard ratios (HR) for cancer incidence in relation to self-reported alcohol consumption (drinks/week) among 225,805 participants aged ≥45 years (2005–2009) in the New South Wales (NSW) 45 and Up Study, an Australian prospective cohort study (baseline *n* = 267,357). Cumulative absolute risk of cancer to age 85 years was estimated using 0 to <1 drink/week as the comparator. Population attributable fractions were calculated using Australian national alcohol consumption and cancer incidence data, compared to a theoretical minimum risk exposure of no alcohol consumption. Cancer cases and deaths were ascertained through record linkage to the NSW Cancer Registry and NSW Registry of Births Deaths & Marriages to 2019. Participants diagnosed with cancer pre-baseline were excluded.

**Results:**

Over a median 11.4 years, 34,860 cancer cases were recorded. When modelled as a continuous variable, alcohol-related cancer risk increased by 19% for every ten drinks/week increase in consumption (HR: 1.19; 95% confidence interval: 1.15–1.23). By age 85 years, those who consumed >10 drinks/week had an estimated 4.9% higher cumulative absolute risk of an alcohol-related cancer compared to those consuming 0 to <1 drink/week. An estimated 7804 cancer cases (4.6% of all cancer cases) were attributable to alcohol use in 2024.

**Conclusions:**

The proportion of alcohol-attributable cancers in Australia is substantial and somewhat higher than previously estimated.

## Introduction

Cancer accounts for a substantial proportion of the health burden worldwide, and in 2019 was responsible for 18% of the burden of disease in Australia and 10% globally [1]. An important risk factor for cancer is alcohol use, which is known to cause at least seven types of cancer, including cancers of the mouth, pharynx, larynx, oesophagus, colorectum, liver and breast [2–4].

Australia has a history of high prevalence of alcohol use, with 77% of Australians aged ≥14 years reporting that they consumed alcohol in the previous 12 months (2022–2023), down from 84% in 2001 [5]. In the most recent global comparisons, Australia’s annual per capita alcohol consumption was higher than the global average (10.6 L vs. 6.4 L for those aged ≥15 years in 2016), and has remained at approximately 10 L since the early 1990s [6]. In 2020, the Australian National Health and Medical Research Council (NHMRC) guideline to reduce the risk of alcohol-related harm was revised, advising the public that “*healthy men and women should drink no more than 10 standard drinks a week and no more than 4 standard drinks on any one day*” [7, 8]. This represented a reduction from the limit of 2 standard drinks per day recommended in the previous (2009) guidelines. In 2022-23, about one in three (32.3%) adults, and two in five (41.8%) young adults aged 18-24 years drank alcohol at levels exceeding the new guideline limits [9].

Previous reports of alcohol-related cancer risk in Australia—including relative risk, absolute risk, and disease burden (e.g. population attributable fractions; PAFs)—have been estimated in relation to standard drinks or grams of alcohol per day and/or aligned with previous guidelines [10–17]. We aimed to establish the cancer-related harms of consuming alcohol above the new guideline recommended limits, by calculating estimates of risk and burden of disease in Australia for all seven alcohol-related cancer types, in relation to 10 standard drinks per week. Specifically, we aimed to estimate both the relative risk and cumulative absolute risk (up to age 85 years) of cancer in relation to alcohol use, and population attributable fractions. Additionally, we assessed the impact fraction for a hypothetical scenario in which all persons in the population consumed ≤10 drinks per week. This analysis extends our previous report [17], and aimed to re-evaluate the impact of drinking patterns on cancer risk with six additional years of follow-up (i.e. the potential interaction between the total number of drinks consumed and the pattern of consumption across the week).

## Methods

### Study sample

The study sample was a prospective cohort study of 267,357 participants, The Sax Institute’s 45 and Up Study. The study’s methods have been previously described [18, 19]. In summary, using the Services Australia Medicare enrolment database, men and women aged ≥45 years were randomly sampled from the general population of New South Wales (NSW), Australia, between 2005 and 2009. All Australian citizens and permanent residents are included in this database, as well as some temporary residents and refugees. Persons aged ≥80 years and those living in rural or remote areas were oversampled. Participants completed a postal questionnaire (available at: https://www.saxinstitute.org.au/solutions/45-and-up-study/use-the-45-and-up-study/data-and-technical-information/) which contained items on socio-demographic factors, health behaviours, and medical history. The study sample comprised approximately 11% of the NSW population aged ≥45 years. Participants also completed a 5-year follow-up questionnaire between 2012 and 2015 (wave 2 questionnaire; median 5.3 years after baseline; response rate 53%).

Participants with a history of cancer in the NSW Cancer Registry (from 1994) or who self-reported ever having cancer on the baseline questionnaire were excluded from the analyses. Participants were not excluded if they had self-reported non-melanoma skin cancer/melanoma only, as skin lesions are common in Australia and skin cancers are often reported inaccurately [20, 21]. Participants with missing data for alcohol consumption and those with record linkage errors were excluded.

Ethics approval for the 45 and Up Study was conferred by the University of New South Wales Human Research Ethics Committee (reference: HC210602), and for this specific analysis by the NSW Population Health Services Research Ethics Committee (reference: 2014/08/551).

### Record linkage

Probabilistic record linkage of the 45 and Up Study questionnaire data to the NSW Cancer Registry and the NSW Registry of Births Deaths and Marriages (RBDM) was performed by the NSW Ministry of Health’s Centre for Health Record Linkage (CHeReL; http://www.cherel.org.au/). The CHeReL used a best practice approach in privacy preserving record linkage [22], along with the open source probabilistic record linkage software Choice Maker [23]. The probabilistic matching process is highly accurate (false-positive and false-negative rates <0.4%), and a detailed explanation of the linkage process has been published previously [24].

### Cancer incidence

Cancer diagnosis was ascertained from the NSW Cancer Registry to 31 December 2019. Cancer types were classified according to the International Classification of Diseases, version 10 (ICD-10) [25]. Cancer types considered causally related to alcohol consumption according to the International Agency for Research on Cancer [2, 3] were analysed separately and grouped, including cancers of the: mouth and pharynx; oesophagus; larynx; (grouped as cancers of the upper aerodigestive tract); colon; rectum; (grouped as colorectum); liver; and female breast. All alcohol-related cancers combined, as well as all cancers combined were also assessed. In a sensitivity analysis, melanoma and cancers of the stomach, pancreas and lung were also assessed.

### Alcohol consumption

Participant alcohol consumption was ascertained using two questionnaire items at both baseline and follow-up: “*About how many alcoholic drinks do you have each week? One drink* = *a glass of wine, middy of beer or nip of spirits (put “0” if you do not drink, or have less than one drink each week)*” and, “*On how many days each week do you usually drink alcohol?*” [26]. Two independent variables were then derived: (1) to assess the total quantity of alcohol consumed in a week, and (2) to assess pattern of consumption across the week.

The following categories were defined for each of the independent variables at baseline:

(1) Number of drinks per week, (‘Weekly alcohol consumption’): ‘0 to <1’, ‘≥1 to ≤3.5’, ‘>3.5 to ≤10’, ‘>10 to ≤20’, ‘>20 to ≤30’ and ‘>30’ drinks per week. Low-volume drinking ( ≥ 1 to ≤3.5 drinks/week) was used as the reference group rather than 0 to <1 drink per week due to address potential bias from the ‘sick-quitter effect’, whereby non-drinkers may have previously quit drinking due to ill-health, potentially resulting in underestimated risks for heavier drinking [27, 28]. The cut-point of 3.5 drinks/week was selected as this is equivalent to 0.5 drinks/day, which has been described as the upper limit of ‘very light drinking’[29]. The remaining cut-points for this variable were selected to align with multiples of the Australian alcohol consumption guideline to reduce the risk of alcohol-related harm of ≤10 standard drinks per week, where one standard drink contains 10 grams of ethanol [7].

(2) Pattern of drinking: In terms of drinking frequency, a cut-point of 1–3 vs. 4–7 days per week was selected to classify participants as consuming alcohol on less or more than half of the days in a week, and to capture those who may only consume alcohol on weekends, versus those who drink across the week. We have used this classification in previous analyses [17, 30]. Participants who consumed <4 drinks/week were excluded from this analysis because they could not have consumed alcohol on more than 1–3 days/week, and therefore could not be divided into two levels of drinking frequency. In terms of quantity of drinking, three categories were used, with an upper cut-point of 10 drinks per week used to align with the Australian alcohol guidelines. This created a variable with six categories (detailed in Supplementary Material p.3).

Weekly alcohol consumption was also assessed as a continuous variable in log-linear Cox regressions, with hazard ratios indicating the change in risk per ten-drink increase in weekly alcohol consumption (among drinkers only). For the continuous variable, participants within each category of alcohol consumption at baseline were assigned the mean quantity of alcohol consumption reported by these same participants at 5-year follow-up (participants who did not complete the 5-year follow-up questionnaire were excluded from this calculation). This was to allow for regression dilution [31, 32], to reduce the potential for misclassification, and to lessen any impact of outliers on the linear trend.

### Potential confounding variables

Potential confounding variables self-reported in the baseline questionnaire are listed in Supplementary Table 1 and 2. Postcode data was used to derive remoteness of place of residence, with categories based on the Accessibility/Remoteness Index of Australia (ARIA + 2006) [33]. All covariates except for sex had a missing indicator category.

### Additional variables for interaction tests

Postcode data was used to derive area-based socio-economic status, using quintiles of the Socio-Economic Indexes for Areas (SEIFA) Index of Relative Socio-Economic Disadvantage (IRSD) [34].

### Statistical analyses

A descriptive analysis was performed of covariates at baseline in relation to alcohol use, including odds ratios of exceeding 10 drinks per week by covariate categories.

Hazard ratios (HR) and 95% confidence intervals (CI) for incidence of each cancer type in relation to alcohol consumption were estimated using Cox proportional hazard regression models, with age as the underlying time variable [35]. This was performed modelling weekly alcohol consumption both as a categorical variable (using ‘≥1 to ≤3.5’ drinks per week as the reference category) and as a continuous variable (estimating the increase in risk associated with an increase in consumption of 1 drink/week); we have chosen to report hazard ratios associated with increments of 10 drinks/week to align with the upper limit of the Australian weekly alcohol guideline of 10 standard drinks per week. Results were also reported using a dose-response increment of 1 drink/week to allow for the assessment of a per-drink increase in weekly alcohol consumption. Censoring occurred at diagnosis of the cancer type in question, death (ascertained from the NSW RBDM), or at the end of study period (31 December 2019), whichever occurred first. All analyses were adjusted for potential confounders as outlined in Supplementary Table 1. Minimally adjusted regressions (including only sex and age as the underlying time variable) are also reported in the Supplementary Material.

As differences in alcohol-related cancer risk have been reported by sex, smoking status and region of the world [2, 3, 36], statistical interaction tests between alcohol consumption as a continuous variable (per ten drink increase in weekly alcohol consumption) among drinkers and sex (men vs. women), smoking status (never smoking vs. former smoking vs. current smoking), and country of birth (Australia vs. other countries) were performed, with alcohol consumption included as a main-effects categorical variable. Further, as there have been differences in alcohol-related harm reported by socio-economic status [37], two-way statistical interaction tests were also performed between alcohol consumption as a continuous variable among drinkers and socio-economic status (SEIFA IRSD quintiles 1 and 2 [lower] vs. 3, 4 and 5 [higher]). Stratified results were reported where relevant. As there may have been insufficient statistical power when performing interaction tests and stratifications for individual cancer types, the interaction tests were also performed for the outcomes of alcohol-related cancers combined and all cancers combined.

For pattern of drinking, a test for interaction between days per week of drinking and the number of drinks consumed per week (both categorical variables) was conducted. The pattern of drinking analysis was not performed for liver cancer or for the individual cancers making up upper aerodigestive tract cancer due to insufficient case numbers.

The cumulative absolute risk of cancer diagnosis from age 25 to 85 years in Australia in 2024 by sex and level of alcohol consumption was estimated (methods detailed in Supplementary Material p.3).

Population attributable fractions (PAFs) for alcohol consumption and cancer in Australia in 2024 by sex were estimated (methods detailed in Supplementary Material p.5). Briefly, hazard ratios from the 45 and Up Study were applied to alcohol consumption prevalence data from the 2011 to 2012 Australian Health Survey [15, 38] and cancer incidence estimates for 2024 from the Australian Institute of Health and Welfare [39], to estimate PAFs for population alcohol consumption and cancer, compared to a theoretical minimum risk exposure of no alcohol consumption. As it was not possible to estimate hazard ratios for former drinking in the 45 and Up Study, internationally sourced relative risks were used [40]. The potential impact fraction for a scenario in which all persons in the population consuming >10 drinks per week had instead consumed exactly 10 drinks per week (the upper limit of the Australian weekly alcohol guideline) was calculated (methods detailed in Supplementary Material p.6).

Sensitivity analyses were performed excluding the first year of follow-up to assess the possibility of reverse causation. Two sensitivity analyses were performed for the continuous variable analysis: one used the mean number of drinks per week in each alcohol consumption category reported at baseline rather than at 5-year follow-up (to assess any impact of regression dilution), and another used the actual number of drinks per week reported by participants rather than the mean of each alcohol consumption category (to assess any impact of outlier levels of drinking). Pattern of drinking was also assessed using categorisations based on the former Australian alcohol consumption guidelines, to allow comparison with the results of our previous report [17]. In addition to the seven cancer types with ‘convincing’ evidence for a causal relationship with alcohol consumption, sensitivity analyses included cancer types for which there is ‘probable’ (stomach cancer) or ‘limited – suggestive’ (pancreatic cancer, lung cancer and melanoma) [4] evidence of increased risk, to assess the impact on cumulative absolute risk and population attributable fraction estimates if these risk relationships are indeed causal. As body mass may be on the causal pathway between alcohol consumption and cancer risk, meaning that it may not be appropriate to adjust for BMI as a covariate, sensitivity analyses including all covariates except for BMI in regression models were also performed.

The proportional hazards assumption was tested for all Cox regressions. If significant violations were detected, then log-log survival curves were plotted, stratified by the variables in violation. Upon visual inspection, if lines were non-parallel for a covariate, a stratified Cox model was fitted to examine whether HRs deviated. If lines were non-parallel for the exposure variable (alcohol consumption), the model was divided into two age groups which contained equal person-years of follow-up ( < 58 and ≥58 years) to investigate any differences in HRs.

The analyses were performed using SAS 9.4 and STATA 16.0. Secure data access was provided through the Sax Institute’s Secure Unified Research Environment (SURE).

## Results

In total, 225,805 of 267,357 participants (84.5%) were included for analysis after excluding 844 participants (0.3%) who withdrew from the study after baseline, 175 (0.07%) from a pilot study, 5 (0.002%) aged <45 years at baseline, 36,198 (13.5%) with a history of cancer at baseline, 4265 (1.6%) with missing information on overall alcohol consumption, and 65 (0.02%) with record linkage errors. The missing data percentage for each covariate is shown in Supplementary Table 1.

Among included participants, the median follow-up period was 11.4 years, in which 34,860 (15.4%) participants were diagnosed with cancer by 31 December 2019. At baseline, 151,441 (67.1%) participants consumed at least one alcoholic drink per week, and 49,312 (21.8%) consumed >10 drinks per week. Of the 116,206 participants who consumed ≥4 drinks per week, 1175 (1.0%) were excluded from the drinking pattern analyses due to missing information on number of drinking-days per week. Of the 115,031 drinkers included in the drinking pattern analyses, 87,488 (76.1%) consumed alcohol 4–7 days per week, including 39,661 (34.5%) who consumed alcohol every day. The distribution of participants consuming >10 drinks per week varied by many of the covariates assessed (Table 1), including significantly higher odds for those with higher indicators of socio-economic status, those with a current or former history of smoking, and those with high levels of physical activity.

**Table 1:** Socio-demographic and other characteristics by alcohol consumption at baseline in the 45 and Up Study (2005–2009).

		Alcoholic drinks per week					Drinking days per week
Characteristic at baseline	*n*	Mean (SD)	0 to ≤ 10 (%)	>10 (%)	OR > 10 drinks/week (95% CI)	*p*	Mean (SD)
All participants	225,805	7.0 (9.7)	78.2	21.8	–	–	2.9 (2.7)
**Sex**						**<0.001**	
Men	104,228	10.0 (12.0)	67.3	32.7	1.00		3.4 (2.7)
Women	121,577	4.5 (6.3)	87.5	12.5	0.29 (0.29–0.30)	<0.001	2.4 (2.6)
**Age (years)**						**<0.001**	
45–54	71,759	7.2 (9.9)	78.7	21.3	1.00		2.7 (2.5)
55–64	75,223	7.6 (10.2)	76.2	23.8	1.09 (1.06–1.12)	<0.001	3.0 (2.7)
65–74	46,337	7.1 (9.7)	76.9	23.1	1.00 (0.97–1.03)	0.80	3.1 (2.9)
≥75	32,081	5.3 (7.9)	83.2	16.8	0.66 (0.64–0.68)	<0.001	2.7 (3.0)
**Remoteness of residence (ARIA** + **)**						**<0.001**	
Major cities	117,435	6.6 (9.2)	79.6	20.4	1.00		2.8 (2.7)
Inner regional	78,669	7.4 (10.0)	76.6	23.4	1.22 (1.19–1.24)	<0.001	3.0 (2.7)
Outer regional	23,269	7.5 (10.8)	76.7	23.3	1.19 (1.15–1.24)	<0.001	2.9 (2.8)
Remote or very remote	2,158	8.0 (11.3)	75.3	24.7	1.29 (1.16–1.43)	<0.001	2.9 (2.8)
**Highest level of education**						**<0.001**	
No school certificate/other qualifications	25,173	5.7 (10.1)	82.8	17.2	1.00		2.2 (2.7)
School/intermediate certificate	48,936	6.3 (9.4)	80.9	19.1	1.29 (1.24–1.34)	<0.001	2.6 (2.7)
Higher school or leaving certificate	22,178	7.4 (10.1)	76.6	23.4	1.40 (1.33–1.46)	<0.001	2.9 (2.7)
Trade/apprenticeship	24,681	9.4 (12.2)	70.0	30.0	1.37 (1.31–1.43)	<0.001	3.2 (2.8)
Certificate/diploma	47,466	6.7 (8.9)	79.2	20.8	1.22 (1.17–1.27)	<0.001	2.9 (2.7)
University degree or higher	54,033	7.4 (8.8)	76.7	23.3	1.29 (1.23–1.34)	<0.001	3.2 (2.6)
**Household income**^**a**^						**<0.001**	
<$20,000	41,584	5.5 (10.0)	83.5	16.5	1.00		2.2 (2.7)
≥$20,000 to <$40,000	38,621	7.0 (10.1)	78.4	21.6	1.31 (1.27–1.36)	<0.001	2.9 (2.8)
≥$40,000 to <$70,000	41,239	7.8 (10.0)	75.6	24.4	1.49 (1.44–1.55)	<0.001	3.1 (2.7)
≥$70,000	56,873	8.7 (9.7)	71.8	28.2	1.76 (1.70–1.82)	<0.001	3.4 (2.5)
**Health insurance status**						**<0.001**	
None	35,335	7.2 (11.0)	78.0	22.0	1.00		2.6 (2.7)
Health care concession card	38,124	5.8 (10.4)	82.6	17.4	0.80 (0.77–0.83)	<0.001	2.2 (2.7)
DVA white or gold card	3368	7.1 (10.9)	78.1	21.9	1.02 (0.93–1.11)	0.70	3.0 (3.0)
Private health insurance—without extras	32,350	7.1 (9.2)	77.6	22.4	1.05 (1.01–1.09)	0.02	3.1 (2.7)
Private health insurance - with extras	112,588	7.4 (9.1)	76.8	23.2	0.82 (0.75–0.90)	<0.001	3.1 (2.7)
**Married/living with partner**						**<0.001**	
No	54,485	6.2 (10.5)	81.9	18.1	1.00		2.4 (2.7)
Yes	170,012	7.3 (9.5)	77.0	23.0	1.10 (1.08–1.13)	<0.001	3.0 (2.7)
**Country of birth**						**<0.001**	
Australia	168,998	7.3 (10.0)	77.0	23.0	1.00		2.9 (2.7)
Canada/Ireland/NZ/UK/USA	28,271	7.8 (9.8)	75.1	24.9	1.08 (1.04–1.10)	<0.001	3.3 (2.7)
Other country	26,773	4.2 (7.3)	88.7	11.3	0.39 (0.37–0.40)	<0.001	2.1 (2.6)
**SEIFA IRSD**						**<0.001**	
Quintile 1 (most disadvantaged)	36,133	6.5 (10.6)	80.2	19.8	1.00		2.4 (2.7)
Quintile 2	36,928	7.0 (10.2)	78.4	21.6	1.11 (1.07–1.14)	<0.001	2.7 (2.7)
Quintile 3	33,290	6.8 (9.4)	78.8	21.2	1.06 (1.03–1.10)	<0.001	2.8 (2.7)
Quintile 4	29,658	7.1 (9.3)	78.0	22.0	1.11 (1.07–1.15)	<0.001	3.0 (2.7)
Quintile 5 (least disadvantaged)	35,900	7.5 (8.8)	75.9	24.1	1.24 (1.20–1.28)	<0.001	3.3 (2.6)
**Smoking status**						**<0.001**	
Never	129,347	5.1 (7.4)	85.8	14.2	1.00		2.5 (2.6)
Former	78,927	9.6 (11.1)	67.5	32.5	2.55 (2.50–2.61)	<0.001	3.5 (2.7)
Current	16,824	10.0 (14.2)	69.4	30.6	2.38 (2.29–2.47)	<0.001	2.9 (2.8)
**Body mass index**^**b**^						**<0.001**	
Underweight	2,734	5.4 (9.1)	84.3	15.7	0.90 (0.81–1.00)	0.06	2.5 (2.8)
Healthy weight	76,976	6.6 (8.8)	79.6	20.4	1.00		3.0 (2.7)
Overweight	82,751	7.8 (10.0)	74.9	25.1	1.05 (1.03–1.08)	<0.001	3.1 (2.7)
Obese	46,825	6.7 (10.7)	79.8	20.2	0.86 (0.84–0.89)	<0.001	2.4 (2.6)
**Physical activity (min/week)**^**c**^						**<0.001**	
Inactive (0)	10,950	5.7 (10.9)	83.1	16.9	1.00		2.1 (2.7)
Insufficient ( > 0 to <150)	36,230	6.1 (9.6)	82.1	17.9	1.01 (0.96–1.07)	0.66	2.5 (2.7)
Sufficient ( ≥ 150 to <300)	34,899	6.8 (9.6)	79.4	20.6	1.20 (1.13–1.27)	<0.001	2.8 (2.7)
High ( ≥ 300)	137,522	7.5 (9.7)	76.3	23.7	1.47 (1.40–1.55)	<0.001	3.1 (2.7)

Variables obtained from the 45 and Up Study baseline questionnaire (https://www.saxinstitute.org.au/solutions/45-and-up-study/use-the-45-and-up-study/data-and-technical-information/). Missing category for each variable not shown. Odds ratios were adjusted for sex and age.

*ARIA +* Accessibility and Remoteness Index of Australia, *CI* Confidence Interval, *DVA* Department of Veterans’ Affairs, *IRSD* Index of Relative Socio-Economic Disadvantage, *NZ* New Zealand, *OR* Odds Ratio, *SD* Standard Deviation, *SEIFA* Socio-Economic Indexes for Areas, *UK* United Kingdom, *USA* United States of America.

^a^Pre-tax annual household income from all sources in Australian dollars.

^b^Underweight: <18.5 kgm^–2^, healthy weight: ≥18.5 to <25 kgm^–2^, overweight: ≥25 to <30 kgm^–2^, obese: ≥30 kgm^–2^.

^c^Weekly physical activity time, with each minute of walking or moderate physical activity counted as 1 min, and each minute of vigorous physical activity counted as 2 min, according to the Australian physical activity guidelines [65].

Bold *p*-values indicate test of significance in relation to variable, non-bold *p*-values indicate test of significance inrelation to individual variable categories.

Compared to those consuming ≥1 to ≤3.5 drinks per week, risk was significantly increased for cancers of the colorectum (HR: 1.21; 95% CI: 1.08–1.35), colon (1.20; 1.04–1.37) and breast (1.21; 1.08–1.35) among participants consuming >10 to ≤20 drinks per week, upper aerodigestive tract (1.39; 1.09–1.75) and mouth and pharynx (1.62; 1.21–2.17) among participants consuming >20 to ≤30 drinks per week, and oesophagus (2.58; 1.58–4.21) and liver (2.89; 1.88–4.44) among participants consuming >30 drinks per week (Table 2). Risk of both alcohol-related cancers combined (1.14; 1.06–1.23) and all cancers combined (1.06; 1.02–1.10) was significantly increased for participants who consumed >10 to ≤20 drinks per week compared to those consuming ≥1 to ≤3.5 drinks per week. For those consuming >3.5 to ≤10 drinks per week, point estimates were not significantly different to those consuming ≥1 to ≤3.5 drinks per week, except for a significant inverse association for laryngeal cancer (see Table 2).

**Table 2:** Hazard ratios (HR) and 95% confidence intervals (CI) of cancer risk by alcohol consumption in the 45 and Up Study (2005–2019).

			HR drinks per week (95% CI)									
Cancer type (ICD-10 code)	*n* cases	Age- and sex-standardised rate^a^	0 to < 1	≥1 to ≤ 3.5	>3.5 to ≤ 10	>10 to ≤ 20	>20 to ≤ 30	>30	*p*^b^	n cases among drinkers	HR per 10 drink increase per week (95% CI)	*p*_trend_^c^
Upper aerodigestive tract (C00-15;32)	1324	54.9	0.92 (0.77–1.11)	1.00	0.98 (0.82–1.18)	1.06 (0.86–1.30)	1.39 (1.09–1.75)	2.20 (1.71–2.82)	<0.001	972	1.27 (1.18–1.37)	<0.001
- Mouth and pharynx (C00-14)	825	34.1	0.82 (0.65-1.04)	1.00	1.02 (0.81–1.28)	1.13 (0.88–1.47)	1.62 (1.21–2.17)	2.12 (1.53–2.94)	<0.001	621	1.27 (1.16-1.39)	<0.001
- Oesophagus (C15)	370	15.4	1.44 (1.00–2.08)	1.00	1.15 (0.79–1.68)	1.05 (0.69–1.60)	1.19 (0.72–1.96)	2.58 (1.58–4.21)	<0.001	248	1.29 (1.11–1.49)	<0.001
- Larynx (C32)	136	5.5	0.57 (0.33–1.01)	1.00	0.55 (0.31–0.97)	0.74 (0.42–1.33)	0.76 (0.38–1.51)	1.63 (0.85–3.13)	0.007	108	1.22 (0.99-1.50)	0.06
Colorectum (C18-20)	4261	183.3	0.98 (0.89–1.08)	1.00	1.00 (0.91–1.11)	1.21 (1.08–1.35)	1.31 (1.13–1.51)	1.49 (1.24–1.79)	<0.001	2,889	1.16 (1.11–1.22)	<0.001
- Colon (C18)	3040	132.1	1.02 (0.91–1.14)	1.00	1.03 (0.91–1.16)	1.20 (1.04–1.37)	1.36 (1.14–1.61)	1.58 (1.27–1.97)	<0.001	2,012	1.18 (1.11–1.25)	<0.001
- Rectum (C19-20)	1271	52.6	0.86 (0.72–1.03)	1.00	0.92 (0.77–1.11)	1.21 (0.99–1.48)	1.21 (0.94–1.56)	1.35 (1.00–1.84)	<0.001	914	1.15 (1.06–1.25)	0.001
Liver (C22)	412	17.3	1.31 (0.95–1.81)	1.00	0.87 (0.61–1.24)	0.89 (0.59–1.32)	1.36 (0.88–2.13)	2.89 (1.88–4.44)	<0.001	258	1.46 (1.28–1.68)	<0.001
Breast (C50^d^)	4609	337.6	0.95 (0.87–1.03)	1.00	1.07 (0.98–1.17)	1.21 (1.08–1.35)	1.34 (1.11–1.61)	1.13 (0.72–1.77)	<0.001	2,891	1.18 (1.09–1.28)	<0.001
**Alcohol-related cancers combined (**^**e**^**)**	10,471	430.6	0.96 (0.90–1.02)	1.00	1.02 (0.96–1.08)	1.14 (1.06–1.23)	1.31 (1.19–1.44)	1.73 (1.53–1.96)	<0.001	6,923	1.19 (1.15–1.23)	<0.001
**All cancers combined (**^**f**^**)**	34,860	1530.9	0.99 (0.96–1.02)	1.00	1.00 (0.97–1.04)	1.06 (1.02–1.10)	1.03 (0.98–1.08)	1.15 (1.08–1.23)	<0.001	24,133	1.05 (1.03–1.07)	<0.001

All models were adjusted for the covariates: remoteness, education, household income, health insurance status, partner status, country of birth, smoking status and intensity, body mass index and physical activity; additional covariates included: sex, fruit intake, vegetable intake and aspirin use for the upper aerodigestive tract cancer and oesophageal cancer models; sex, fruit intake and vegetable intake for the mouth and pharynx cancer and larynx cancer models; sex, fruit intake, vegetable intake, fibre intake, red meat intake, processed meat intake, menopausal hormone therapy use, aspirin use and bowel screening history for the colorectum cancer, colon cancer and rectum cancer models; sex and hormonal contraceptive use for the liver cancer model; parity and age at first birth, breastfeeding duration, menopausal status, hormonal contraceptive use, menopausal hormone therapy use and breast screening history for the breast cancer model; sex, fruit intake, vegetable intake, fibre intake, red meat intake, processed meat intake, parity and age at first birth, breastfeeding duration, menopausal status, hormonal contraceptive use, menopausal hormone therapy use, aspirin use, bowel screening history and breast screening history for the alcohol-related cancers combined model; and sex, fruit intake, vegetable intake, fibre intake, red meat intake, processed meat intake, time spent outdoors, skin tone, parity and age at first birth, breastfeeding duration, menopausal status, hormonal contraceptive use, menopausal hormone therapy use, aspirin use, bowel screening history, breast screening history and prostate screening history for the all cancers combined model. Cancer cases do not sum to totals as some participants were diagnosed with two or more primary cancers.

^a^Standardised rate per 100,000 person-years, standardised by age and sex to the 2006 New South Wales population aged ≥45 years.

^b^*p* heterogeneity.

^c^Linear trend in categories calculated among drinkers only, where participants within each category of alcohol consumption at baseline were assigned the mean level of alcohol consumption they reported at first wave follow-up (median 5.3 years after baseline).

^d^Breast cancer in women only.

^e^C00-15;18-20;22;32;50, including breast cancer in women only.

^f^C00-97;D45-46;47.1;47.3-47.5. ICD-10, International Classification of Diseases, version 10.

In the continuous variable analysis among drinkers, risk significantly increased with increasing levels of alcohol use for all cancer types examined except for laryngeal cancer (Table 2). For every ten drinks per week, the risk of alcohol-related cancers combined increased by 19%, and the risk of all cancers combined increased by 5%. Results using a dose-response increment of 1 drink/week rather than 10 drinks/week are shown in Supplementary Table 3.

Significant statistical interactions were observed for cancer risk in the continuous variable analysis per ten drink increase in weekly alcohol consumption by sex for liver cancer and alcohol-related cancers combined, with smoking status in relation to oesophageal cancer and alcohol-related cancers combined, and with country of birth in relation to colon cancer (Supplementary Table 4). There were no statistically significant interactions for socio-economic status.

**Fig. 1:**
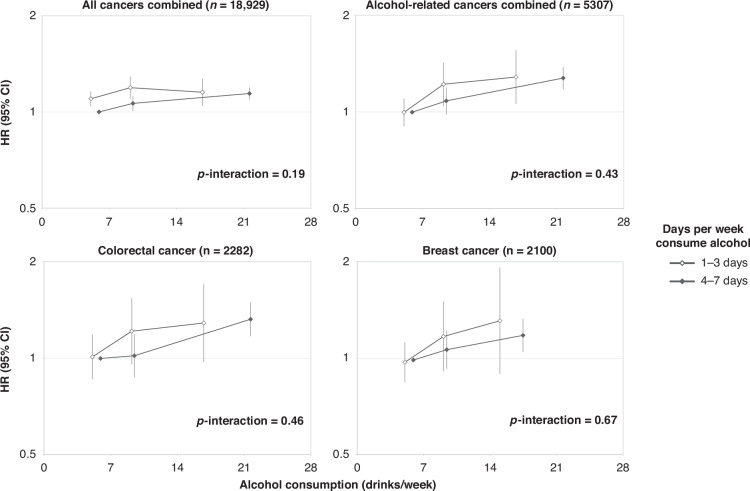
Hazard ratios (HR) and 95% confidence intervals (CI) of cancer risk by pattern of drinking among participants consuming ≥ 4 drinks per week in the 45 and Up Study (2005–2019). Models were adjusted for cancer-specific covariates as listed in Supplementary Table 1. Reference category: ≥4 to <7 drinks per week, consumed on 4–7 days per week. Point estimates plotted at mean intake for each of the three levels of overall alcohol consumption ( ≥ 4 to ≤7 drinks per week, >7 to ≤10 drinks per week, and >10 drinks per week). *p*_interaction_ is for test of interaction between days per week and drinks per week. Breast cancer in women only.

Cancer risk did not vary significantly in relation to drinking pattern (Fig. 1 and Supplementary Table 5). Specifically, there were no statistically significant interactions between days per week of drinking and the number of drinks consumed per week for any of the cancer outcomes examined. Results did not differ to those using categorisation based on the former Australian alcohol consumption guidelines (Supplementary Table 6).

By age 85 years, the absolute risk of an alcohol-related cancer among those consuming >10 drinks per week was 15.7% for men and 25.0% for women, compared to 10.8% for men and 20.1% for women among those consuming 0 to <1 drink per week (Supplementary Table 7). This represented an absolute risk increase of 4.9% in both men and women. The largest contributing individual cancers to this increase in risk were breast cancer in women, and colorectal cancer in men. For those consuming ≥1 to ≤10 drinks per week, the absolute risk of an alcohol-related cancer by age 85 years was 11.8% for men and 21.7% for women.

**Fig. 2:**
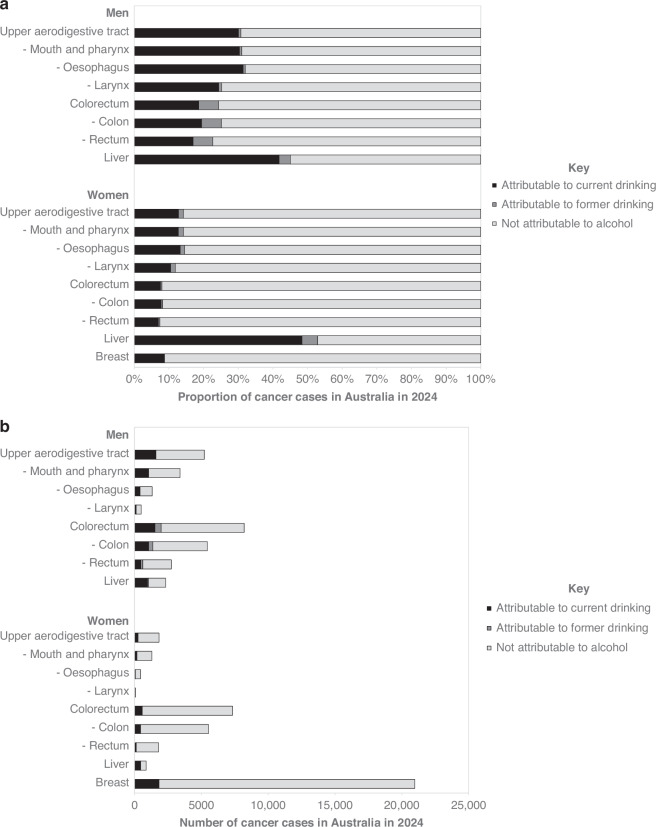
Attributable fraction (**a**) and attributable number (**b**) of cancer cases caused by alcohol consumption in the Australian population in 2024, derived using risk estimates from the 45 and Up Study (2005–2019) for current drinking and international data for former drinking. Hazard ratios used in calculations for the 45 and Up Study were derived from total alcohol consumption as a log-linear variable among participants consuming ≥1 drink per week. For each age group used in the calculation, cancer incidence was attributed to alcohol consumption approximately 10 years earlier in the 2011–2012 Australian Health Survey. Breast cancer in women only.

The population attributable fractions for alcohol consumption and cancer incidence in the Australian population in 2024 are shown in Table 3 and Fig. 2. Of the 169,478 expected cancer cases, 7128 (4.2%) were estimated to be attributable to current alcohol consumption, or 4.4% in men and 4.0% in women. By cancer type, estimates ranged from 8.8% for breast cancer to 43.6% for liver cancer. When cancers attributable to former drinking were accounted for, a total of 7804 (4.6%) cancer cases were estimated to be attributable to alcohol consumption, or 5.0% in men and 4.1% in women. If all persons in the population consuming >10 drinks per week had instead consumed exactly 10 drinks per week (the upper limit of the Australian weekly alcohol guideline), 3733 cancer cases could have been prevented in 2024, resulting in a potential impact fraction for all cancer of 2.2% (Supplementary Table 8).

**Table 3:** Population attributable fractions for cancer caused by alcohol consumption in the Australian population in 2024, derived using risk estimates from the 45 and Up Study (2005–2019) for current drinking and international data for former drinking.

	Men		Women		Persons	
Cancer type (ICD-10 code)	n alcohol-attributable cases/n total cases	PAF (%)	n alcohol-attributable cases/n total cases	PAF (%)	n alcohol-attributable cases/n total cases	PAF (%)
***Cancer cases attributable to current alcohol consumption***						
Upper aerodigestive tract (C00-15;32)	1572/5230	30.1	236/1843	12.8	1808/7073	25.6
- Mouth and pharynx (C00-14)	1035/3411	30.3	166/1304	12.8	1201/4715	25.5
- Oesophagus (C15)	416/1323	31.4	61/462	13.3	477/1785	26.7
- Larynx (C32)	121/496	24.4	8/77	10.5	129/573	22.5
Colorectum (C18-20)	1528/8205	18.6	557/7337	7.6	2084/15,542	13.4
- Colon (C18)	1059/5448	19.4	431/5539	7.8	1490/10,987	13.6
- Rectum (C19-20)	468/2757	17.0	126/1798	7.0	594/4555	13.0
Liver (C22)	977/2336	41.8	422/872	48.4	1399/3208	43.6
Breast (C50^a^)	–/–	–	1837/20,973	8.8	1837/20,973	8.8
**Alcohol-related cancers combined (C00-15;18-20;22;32;50**^**a**^**)**	4076/23,976	17.0	3051/38,362	8.0	7128/62,338	11.4
**All cancers combined (C00-97;D45-46;47.1;47.3-47.5)**	4076/93,504	4.4	3051/75,974	4.0	7128/169,478	4.2
***Cancer cases attributable to former alcohol consumption***						
Upper aerodigestive tract (C00-15;32)	37/5230	0.7	25/1843	1.4	62/7073	0.9
- Mouth and pharynx (C00-14)	24/3411	0.7	19/1304	1.4	43/4715	0.9
- Oesophagus (C15)	8/1323	0.6	6/462	1.2	14/1785	0.8
- Larynx (C32)	4/496	0.8	1/77	1.3	5/573	0.9
Colorectum (C18-20)	468/8205	5.7	31/7337	0.4	499/15,542	3.2
- Colon (C18)	311/5448	5.7	24/5539	0.4	335/10,987	3.1
- Rectum (C19-20)	157/2757	5.7	7/1798	0.4	164/4555	3.6
Liver (C22)	75/2336	3.2	40/872	4.5	115/3208	3.6
Breast (C50^a^)	–/–	–	0/20,973	0.0	0/20,973	0.0
**Alcohol-related cancers combined (C00-15;18-20;22;32;50**^**a**^**)**	580/23,976	2.4	96/38,362	0.3	677/62,338	1.1
**All cancers combined (C00-97;D45-46;47.1;47.3-47.5)**	580/93,504	0.6	96/75,974	0.1	677/169,478	0.4
***Total cancer cases attributable to alcohol consumption***						
Upper aerodigestive tract (C00-15;32)	1609/5230	30.8	261/1843	14.2	1870/7073	26.4
- Mouth and pharynx (C00-14)	1059/3411	31.1	185/1304	14.2	1244/4715	26.4
- Oesophagus (C15)	424/1323	32.1	67/462	14.5	491/1785	27.5
- Larynx (C32)	125/496	25.2	9/77	11.8	134/573	23.4
Colorectum (C18-20)	1996/8205	24.3	588/7337	8.0	2584/15,542	16.6
- Colon (C18)	1371/5448	25.2	455/5539	8.2	1826/10,987	16.6
- Rectum (C19-20)	625/2757	22.7	133/1798	7.4	758/4555	16.6
Liver (C22)	1052/2336	45.0	462/872	53.0	1514/3208	47.2
Breast (C50^a^)	–/–	–	1837/20,973	8.8	1837/20,973	8.8
**Alcohol-related cancers combined (C00-15;18-20;22;32;50**^**a**^**)**	4657/23,976	19.4	3148/38,362	8.2	7804/62,338	12.5
**All cancers combined (C00-97;D45-46;47.1;47.3-47.5)**	4657/93,504	5.0	3148/75,974	4.1	7804/169,478	4.6

Hazard ratios used in calculations for the 45 and Up Study were derived from total alcohol consumption as a log-linear variable among participants consuming ≥1 drink per week. For each age group used in the calculation, cancer incidence was attributed to alcohol consumption approximately 10 years earlier in the 2011-2012 Australian Health Survey. ‘n excess cases’ refers to cancer cases attributable to alcohol consumption out of the total number of cases, ‘n cases’. Cancer cases may not sum to totals due to rounding.

^a^Breast cancer in women only. ICD-10, International Classification of Diseases, version 10. PAF, Population Attributable Fraction.

Results did not materially differ when the year of follow-up was excluded (Supplementary Tables 3, 9, 10). Using the mean number of drinks per week of each alcohol consumption category reported at baseline (rather than at 5-year follow up to account for regression dilution) and using the actual number of drinks per week reported by participants (rather than the mean of each drinking category) both attenuated hazard ratios for all outcomes in the continuous variable analysis, while the risk of laryngeal cancer became significantly increased due to a more narrow confidence interval (Supplementary Table 3).

For cancer types with probable or suggestive evidence for increased risk, there was a statistically significant relationship in the continuous variable analysis per ten drink increase in weekly alcohol consumption for lung cancer, but not for stomach cancer, pancreatic cancer or melanoma (Supplementary Tables 11 and 12). A significant statistical interaction was observed for cancer risk in the continuous variable analysis per ten drink increase in weekly alcohol consumption by smoking status for stomach cancer, but not for any of the other additional cancer types, and there were no statistically significant interactions for sex, country of birth or socio-economic status (Supplementary Table 13). Findings for cumulative absolute risk of alcohol-related cancer for these additional cancer types are presented in Supplementary Table 14. When these additional cancer types were included in the PAF calculations, the percentage of cancer cases attributable to alcohol consumption increased from 4.2% to 5.4% when former drinking was not included and from 4.6% to 5.9% when former drinking was included (Supplementary Table 15).

Minimally adjusted hazard ratios are shown in Supplementary Tables 16, 17, 18. Results did not materially differ when the regressions did not include BMI as a covariate (Supplementary Tables 17, 19, 20). The proportional hazards assumption was violated in some cases (see Supplementary Material p.7 and Supplementary Tables 21 and 22).

## Discussion

In this Australian cohort, risk of cancer increased with increasing alcohol use. For every ten drinks per week, the risk of upper aerodigestive tract cancer increased by 27%, colorectal cancer by 16%, liver cancer by 46%, breast cancer by 18%, alcohol-related cancers combined by 19%, and all cancers combined by 5%. While cancer risk in relation to alcohol is on a continuum, with no specific threshold indicating a lack of risk, consumption of alcohol above the National guideline limit of 10 drinks per week significantly increased the risk of alcohol-related cancers, compared to low-volume drinking ( ≥ 1 to ≤3.5 drinks per week). By age 85 years, men and women who consumed >10 drinks per week were estimated to have 4.9% higher cumulative absolute risk of an alcohol-related cancer compared to those who had 0 to <1 drink/week, with the largest contributing individual cancers being breast cancer in women and colorectal cancer in men. Overall, an estimated 7,804 cases of cancer (4.6% of all cases) were attributable to alcohol consumption in Australia in 2024, and there would be 3,733 fewer cancer cases (2.2% of all cases) if the population of Australia adhered to the guidelines of 10 drinks per week or fewer.

Our updated evaluation, with 6 additional years of follow-up and 17,528 more participants diagnosed with cancer than our previous analysis [17] demonstrates higher relative risks for cancer in relation to alcohol consumption than previously estimated. For example, when modelled as a continuous variable, the risk of alcohol-related cancers combined increased by 10% for every *seven* drinks per week in the previous analysis, and by 19% for every *ten* drinks per week in the present analysis. Compared to the linear dose-response meta-analyses reported by the World Cancer Research Fund (WCRF) [41], our hazard ratios were higher. For example, the WCRF reported hazard ratios of 1.18 (95% CI: 1.10–1.26), 1.07 (1.05–1.08) and 1.04 (1.02–1.06) for cancers of the upper aerodigestive tract, colorectum and liver, respectively, per the equivalent of *seven* drinks per week. The corresponding hazard ratios in our study per *ten* drinks per week were 1.27 (1.18–1.37), 1.16 (1.11–1.22) and 1.46 (1.28–1.68), respectively. The WCRF reported hazard ratios of 1.05 (1.02–1.08) and 1.09 (1.07–1.12) for pre- and post-menopausal breast cancer, respectively per *seven* drinks per week, compared to our finding of 1.18 (1.09–1.28) for all breast cancer per *ten* drinks per week. In sensitivity analyses we showed that hazard ratios were higher after accounting for regression dilution, meaning that estimates from studies that did not perform this correction may have been weakened by random measurement error in alcohol intake. Findings may also vary by calendar period and from country to country, which is why contemporary local data are important for understanding the burden of alcohol-related cancer in Australia. The use of contemporary hazard ratios from the 45 and Up Study is also a strength of our population attributable fraction estimates, as previous estimates for alcohol-related cancer burden in Australia have used earlier estimates of relative risks based on international data.

The updated analyses did not identify significant variations in cancer risk by drinking pattern, in terms of number of days of drinking per week. Further, in our previous analysis of breast cancer risk there was a marginally significant finding of higher risk among women who consumed alcohol on 1–3 days per week compared to those who consumed alcohol on 4–7 days per week, which was not confirmed using these more comprehensive data. It should be noted however that there still may have been limited statistical power to detect differences by drinking pattern. As reported in our previous analysis, findings for the relationship between patterns of drinking and cancer risk have been mixed, and have been complicated by studies using different methods to define drinking patterns and the fact that many studies do not account for overall alcohol intake, meaning that it is not possible to distinguish any effect of drinking pattern from that of overall alcohol intake [17]. Several studies that have accounted for overall alcohol intake have found significant effects on cancer risk in relation to variously defined patterns of drinking [42–48], but not all [49, 50]. Patterns of drinking may also be defined in terms of stable or changing trajectories in volume of alcohol consumption over a lifetime, and significant differences in cancer risk between different lifetime drinking trajectories have been reported [51, 52]. Further insights into the relationship between patterns of alcohol consumption (including drinking frequency, heavy episodic drinking/number of drinks per drinking occasion, and drinking trajectories over a lifetime) and cancer risk may be gained by comprehensive evidence synthesis of studies that account for overall alcohol consumption when calculating risk.

Our result of 4.9% for the excess cumulative lifetime absolute risk of alcohol-related cancer in men and women consuming >10 drinks per week compared to those consuming <1 drink per week was somewhat higher than that of our previous study, which found a result of 4.4% for men and 5.4% for women consuming >14 drinks per week (a greater level of drinking based on an older version of the alcohol guidelines) compared to those consuming <1 drink per week. This reflects the slightly higher relative risks found in our updated study. Absolute risk estimates provide added value over relative risks because they are easier for individuals to understand and are an effective method for communicating risk to the public [53]. Relative risks, by contrast, require knowledge of the underlying incidence rate in order to interpret their potential impact. For example, although the largest hazard ratios in our study were for liver and oesophageal cancer, the greatest contributions to excess absolute risk were from breast cancer in women and colorectal cancer in men, due to their higher base incidence. We are aware of two studies world-wide which have previously estimated lifetime absolute risks for alcohol and cancer. One study estimated that consumption of 240 grams of ethanol (24 Australian standard drinks) per week was associated with an increased absolute risk of cancer of 1.9% in non-smoking men and 3.6% in non-smoking women to age 80 years in the United Kingdom in 2010 [54]. The second study estimated that every 10 grams of alcohol per day (7 Australian standard drinks per week) was associated with an excess of 15 cases per 1000 women to age 75 years in developed countries in 2000 [31]. Differences between these findings reflect the use of different quantities of alcohol consumption, relative risks, and ending ages in the calculations, the calculation method used, that population cancer incidence rates vary from country to country, and that the non-smoking population has a lower incidence of cancer compared to the general population.

Our estimate that 4.2% of cancers in Australia in 2024 were attributable to current alcohol consumption is broadly compatible with previously published Australian estimates, which have included 2.8% in 2010 [12], 2.8% in 2013 [55] and 4.1% in 2020 [40], as well as 3.6% of the burden of disease from cancer in 2024 [56]. Estimates have varied for specific cancer types, including for head and neck cancer [12, 57], oesophageal cancer [12, 58], colorectal cancer [12, 59], liver cancer [12], and breast cancer [12, 14]. Reasons for these differences include (1) the differing sources of relative risks including that our relative risks were somewhat higher than earlier estimates, (2) the calculation method used, (3) whether the estimate related to total alcohol consumption (our study and [12, 14, 40, 55, 56]) or for alcohol consumption above a threshold of drinking ( > 2 drinks/day [57, 59] or >3 drinks/day [58]), and (4) the differing study periods used for cancer incidence (2024 in our study vs. 2010 [12], 2013 [55], 2020 [40], 2024 [56], 2017–2026 [14, 57, 59] and 2021–2030 [58]) and alcohol consumption prevalence (2011–2012 in our study vs. 2001 [12, 55], 2010 [40], 1999–2015 [14], 2013–2015 [59], 2017–2018 [57, 58] and 2022–2023 [56]). Our PAF estimates were based on Australian relative risk data and accounted for cancer cases attributable to former drinking, some unique points of difference from previous estimates for Australia.

It was estimated that if all persons in the population consuming >10 drinks per week had instead consumed exactly 10 drinks per week, 3733 cancer cases would have been prevented in 2024 (a potential impact fraction for all cancer of 2.2%). This represents 52% of the estimated 7128 cancer cases attributable to current alcohol consumption in 2024, meaning that 48% of the cancer cases attributable to current alcohol consumption in Australia are attributable to drinking within the NHMRC guideline of 10 drinks per week. This is consistent with results from a previous Australian study, which estimated that 1442 (45%) of the 3208 cancers attributable to current alcohol consumption in 2010 would be prevented if all persons consumed ≤2 drinks per day, although the cut-point was based on an older version of the alcohol guidelines. As well as conveying the importance of those drinking more heavily reducing their consumption, these findings also mean that a sizeable portion of the cancers attributable to alcohol use in Australia could only be prevented if persons consuming within the alcohol guidelines cease or reduce their consumption. Therefore, it should be emphasised that almost half of the burden of alcohol-related cancer in Australia is caused by within-guideline drinking, demonstrating that all Australians who drink alcohol, not just those drinking above the guidelines, have potential to lower their cancer risk by reducing their level of alcohol use.

It should be noted that there are alternative methods for calculating population attributable fractions. Most PAF estimates to date have reported findings relating to the number of cancer cases attributable to alcohol use in a single year [12, 40, 55] or in a set of years [14, 57–59] in the recent past or near future. Another study used simulation modelling to instead estimate the number of cancer cases that could be prevented in Australia over the years 2013–2037 if an intervention occurred in 2012 that caused all population alcohol consumption to cease, or that caused all persons to consume a maximum of 20 g ethanol per day [15]. Importantly, the method accounted for latency time—the time it would take between quitting/reducing alcohol consumption, and when relative risk would begin to decrease and how long it would take to for the relative risk to reach 1 or that of a lower category of alcohol consumption. It was found that approximately 49,500 or 29,600 cancer cases would be prevented over the 25-year study period respectively, under the scenario of a rapid latency time. A further study used the future excess fraction method to estimate lifetime burden of cancer due to alcohol consumption and other risk factors to the year 2098 for the cohort of adults in Australia in 2016 [60]. The study reported that approximately 250,000 cancer cases would be attributable to alcohol consumption over the lifetime of the cohort, which was 3.3% of the total ~7,600,000 cases projected to occur. These methods are complementary to one another, as each approach offers a different perspective of the burden of alcohol consumption today and into the future, and of the potential impact of population interventions.

Another notable finding was that of the four cancer types with probable or suggestive evidence for increased risk with alcohol consumption, increased risk with drinking in the continuous variable analysis was observed for lung cancer, but not for stomach cancer, pancreatic cancer or melanoma. In the categorical variable analysis, participants who consumed 0 to <1 drink/week also had higher risk of lung cancer than those who consumed ≥1 to ≤3.5 drinks/week. According to the WCRF, there is ‘limited – suggestive’ evidence that alcohol consumption is causally related to lung cancer [4], while the International Agency for Research on Cancer concluded that there is inadequate available data to determine whether a causal association exists [3]. It is possible that our findings may represent residual confounding by smoking rather than a causal association.

It was also found that higher alcohol consumption at baseline in the 45 and Up Study cohort was associated with greater levels of physical activity, a finding which has frequently been reported in longitudinal studies and cross-sectional population surveys [61]. Hypotheses to explain this relationship include the excitement-seeking and risk-taking preferences in individuals, the socialisation that occurs with organised sport, and that individuals may try to compensate for the harmful effects of alcohol use through physical activity [61, 62]. As there is evidence that physical activity prevents colorectal cancer and breast cancer [4], any residual confounding from physical activity in our study may have resulted in alcohol-attributable colorectal and breast cancer risk being underestimated.

Strengths of our study include the use of a large prospective cohort study with record linkage and a long period of independent follow-up to generate robust local estimates for alcohol and cancer risk, and the use of these findings to estimate cumulative absolute risk and population attributable fractions for alcohol and cancer in Australia. Our PAF estimates accounted for cancers attributable to former drinking, which have not been accounted for in previous Australian estimates. This is analogous to how cancers attributable to former smoking are normally included in PAF estimates for tobacco smoking in Australia [63–65]. Some limitations of our study should be noted. Firstly, the PAF analyses assumed that cancer risk in relation to alcohol use does not differ by age, enabling cancer cases attributable to alcohol consumption between the ages of 25 and 45 years to be estimated. Secondly, internationally sourced relative risks were used to estimate cancer cases attributable to former drinking, as it was not possible to estimate hazard ratios for former drinking in the 45 and Up Study. Any potential impact on the overall PAF estimate is likely to be minor, as cancers attributable to former drinking were estimated to make up a relatively small portion (approximately 9%) of the total PAF. Other limitations relating to the estimation of hazard ratios in this study include that people categorised as consuming no alcoholic drinks per week included those who never drank and former and occasional (below-weekly) drinkers, that participants in the 45 and Up Study may have health behaviours that differ from the general population and this may result in heavier drinkers being underrepresented in the cohort, and that participants may have underreported their level of intake. Some further limitations to the population attributable fraction and cumulative absolute risk calculations are detailed in the Supplementary Material (p.4-6).

Internationally, there has recently been a renewed focus on alcohol and cancer, with the release of the 2025 U.S. Surgeon General’s Advisory on alcohol and cancer risk [53]. Alcohol use is the third leading cause of preventable cancer in America, after tobacco and overweight and obesity [66]. Despite this, only 45% of the population was aware that alcohol consumption is a risk factor for cancer in 2019, lower than for many other established cancer risk factors [67]. The Surgeon General issued several calls to action, including for a cancer warning label on alcoholic beverages, to reassess alcohol consumption guidelines to account for alcohol and cancer risk, for expanded education efforts, for increased alcohol screening, education, interventions and referrals in clinical settings, and to incorporate alcohol reduction strategies into population cancer prevention initiatives and plans. These align with the World Health Organization’s Global Alcohol Action Plan 2022-2030, which includes recommendations for implementing high-impact strategies and interventions outlined in its 2018 SAFER initiative, raising awareness of alcohol-related harms and policy measures in the general public and decision makers, increased national and international coordination to address alcohol-related harms, and strengthening the capacity and resources of all countries to generate data on alcohol use, alcohol-related harm and implementation of alcohol control measures, and to implement strategies and interventions to reduce alcohol-related harms [68, 69].

In Australia, there are similar levels of awareness to the United States, with a 2020 survey finding that 51% of Australians are aware that alcohol use can cause cancer overall [70], and a 2019 survey finding awareness for specific cancer types as low as 16% for breast cancer and 29% for mouth and throat cancer [71]. The National Alcohol Strategy was developed by the Australian Commonwealth Government in consultation with State and Territory governments, and aims to reduce harmful alcohol consumption by 10% between 2019 and 2028 [72]. Between 2019 and 2022–2023, the prevalence of drinking above the NHMRC guideline to reduce the risk of alcohol-related harm in Australians aged ≥14 years decreased from 32.0% to 30.7% [9]. This was not a statistically significant decline, so it is unclear whether Australia is on track to meet the aim of reducing harmful alcohol consumption by 10% by 2028. The National Alcohol Strategy has four priority areas of focus, which include improving community safety and amenity, managing availability, price and promotion, supporting individuals to obtain help and systems to respond, and promoting healthier communities, with the latter priority area relating directly to increasing community awareness of the harms of alcohol use, including cancer. Our findings contribute to the evidence base for the long-term harms of alcohol use in Australia, and can be used to justify the case for these policies and interventions as part of the cancer control continuum, and inform the potential health gains of their implementation.

In conclusion, we have estimated relative risks, absolute risks, and population attributable fractions for cancer caused by alcohol use in Australia in relation to current alcohol guidelines. We find that 4.6% of cancers in Australia in 2024 were attributable to alcohol consumption—somewhat higher than previously estimated. We also find risk to be on a continuum of increasing cancer risk with increasing alcohol use, and that a sizeable portion of the burden of alcohol-related cancer in Australia occurs at levels of drinking below the national guideline of 10 drinks per week. This demonstrates that all Australians who drink alcohol—and the population as a whole—have potential to benefit by reducing their level of drinking. There is a clear need for public health interventions in Australia to focus on the longer-term harms of alcohol use, and on middle aged and older persons, in addition to the short-term harms in younger persons that are often the target of public health campaigns. Our study supports actions aiming to lower alcohol consumption in Australia, ultimately reducing the burden of cancer.

## Supplementary information

Supplementary Material

## Data Availability

The data that support the findings of this study are available from the Sax Institute (https://www.saxinstitute.org.au/solutions/45-and-up-study/).

## Code availability

Code not available.
