# Supplementary material for: Alcohol-attributable cancer risk and burden estimates for Australia’s updated alcohol consumption guidelines
Source: Br J Cancer. 2026 Apr 10;135(1):104–17. doi: 10.1038/s41416-026-03403-3 (PMC13270007; doi:10.1038/s41416-026-03403-3)
Supplement: Supplementary file 1 — Supplementary Material [file 41416_2026_3403_MOESM1_ESM.docx]

**Supplementary Material**

**Article title:** Alcohol-attributable cancer risk and burden estimates for Australia’s updated alcohol consumption guidelines.

**Journal:** British Journal of Cancer.

**Author names:** Peter Sarich, Karen Canfell, Sam Egger, Emily Banks, Grace Joshy, Lyndal Wellard-Cole, Clare Hughes, Nehmat Houssami, Paul Grogan, Marianne F Weber.

**Affiliation and email address of the corresponding author, Peter Sarich:** Cancer Elimination Collaboration, Sydney School of Public Health, Faculty of Medicine and Health, The University of Sydney. Email address: peter.sarich@sydney.edu.au

**Summary:** This supplementary file contains additional methods for pattern of drinking, cumulative absolute risk and population attributable fractions, additional results for proportional hazards assumption violations, and supplementary tables and figures.

**Table of contents**

Additional methods3

Additional results7

Supplementary Table 1. Covariates included in models by cancer type in the 45 and Up Study (2005-2019)8

Supplementary Table 2. Categories used in model covariates 9

Supplementary Table 3. Additional analyses for hazard ratios (HR) and 95% confidence intervals (CI) of cancer risk per ten drink increase in weekly alcohol consumption among drinkers in the 45 and Up Study (2005-2019)10

Supplementary Table 4. Interaction tests and stratifications for hazard ratios (HR) and 95% confidence intervals (CI) of cancer risk per ten drink increase in weekly alcohol consumption among drinkers in the 45 and Up Study (2005-2019)10

Supplementary Table 5. Hazard ratios (HR) and 95% confidence intervals (CI) of cancer risk by pattern of drinking among participants consuming ≥4 drinks per week in the 45 and Up Study (2005-2019)11

Supplementary Table 6. Hazard ratios (HR) and 95% confidence intervals (CI) of cancer risk by pattern of drinking among participants consuming ≥4 drinks per week in the 45 and Up Study (2005-2019), using categorisation based on the former Australian alcohol consumption guidelines in a previous analysis of the 45 and Up Study11

Supplementary Table 7. Cumulative absolute risk (%) of cancer diagnosis from age 25 to 85 years in Australia in 2024 by sex and level of alcohol consumption using hazard ratios from the 45 and Up Study (2005-2019)12

Supplementary Table 8. Population attributable fractions for cancer caused by alcohol consumption in the Australian population in 2024, if all persons who consumed >10 drinks per week reduced their consumption to 10 drinks per week, derived using risk estimates from the 45 and Up Study (2005-2019)14

Supplementary Table 9. Hazard ratios (HR) and 95% confidence intervals (CI) of cancer risk by alcohol consumption, with exclusion of the first year of follow-up, in the 45 and Up Study (2005-2019)15

Supplementary Table 10. Hazard ratios (HR) and 95% confidence intervals (CI) of cancer risk by pattern of drinking among participants consuming ≥4 drinks per week, with exclusion of the first year of follow-up, in the 45 and Up Study (2005-2019)16

Supplementary Table 11. Hazard ratios (HR) and 95% confidence intervals (CI) of cancer risk by alcohol consumption for additional cancer types in the 45 and Up Study (2005-2019)16

Supplementary Table 12. Hazard ratios (HR) and 95% confidence intervals (CI) of cancer risk by pattern of drinking among participants consuming ≥4 drinks per week for additional cancer types in the 45 and Up Study (2005-2019)16

Supplementary Table 13. Interaction tests and stratifications for hazard ratios (HR) and 95% confidence intervals (CI) of cancer risk per ten drink increase in weekly alcohol consumption among drinkers for additional cancer types in the 45 and Up Study (2005-2019)17

Supplementary Table 14. Cumulative absolute risk (%) of cancer diagnosis from age 25 to 85 years in Australia in 2024 by sex and level of alcohol consumption for additional cancer types using hazard ratios from the 45 and Up Study (2005-2019)18

Supplementary Table 15. Population attributable fractions for cancer caused by alcohol consumption in the Australian population in 2024 for additional cancer types, derived using risk estimates from the 45 and Up Study (2005-2019) for current drinking and international data for former drinking19

Supplementary Table 16. Hazard ratios (HR) and 95% confidence intervals (CI) of cancer risk by alcohol consumption, with minimal adjustment for covariates in models, in the 45 and Up Study (2005-2019)20

Supplementary Table 17. Hazard ratios (HR) and 95% confidence intervals (CI) of cancer risk per ten drink increase in weekly alcohol consumption among drinkers, with different levels of adjustment for covariates, in the 45 and Up Study (2005-2019)21

Supplementary Table 18. Hazard ratios (HR) and 95% confidence intervals (CI) of cancer risk by pattern of drinking among participants consuming ≥4 drinks per week, with minimal adjustment for covariates in models, in the 45 and Up Study (2005-2019)21

Supplementary Table 19. Hazard ratios (HR) and 95% confidence intervals (CI) of cancer risk by alcohol consumption, without adjustment for body mass index, in the 45 and Up Study (2005-2019)22

Supplementary Table 20. Hazard ratios (HR) and 95% confidence intervals (CI) of cancer risk by pattern of drinking among participants consuming ≥4 drinks per week, without adjustment for body mass index, in the 45 and Up Study (2005-2019)22

Supplementary Table 21. Violations of the proportional hazards assumption in models23

Supplementary Table 22. Cox models stratified by age for models with violations of the proportional hazards assumption for alcohol consumption23

Supplementary material references24

**Additional methods**

*Pattern of drinking*

Six categories were used for the pattern of drinking variable:

- ‘≥4 to ≤7 drinks/week over 4-7 days’ (on average, ≥1 to ≤1.75 drinks per drinking day; reference category)
- ‘>7 to ≤10 drinks/week over 4-7 days’ (on average, >1 to ≤2.5 drinks per drinking day)
- ‘>10 drinks/week over 4-7 days’ (on average, >1.4 drinks per drinking day)
- ‘≥4 to ≤7 drinks/week over 1-3 days’ (on average, ≥1.3 to ≤7 drinks per drinking day)
- ‘>7 to ≤10 drinks/week over 1-3 days’ (on average, >2.3 to ≤10 drinks per drinking day)
- ‘>10 drinks/week over 1-3 days’ (on average, >3.3 drinks per drinking day).

*Cumulative absolute risk*

The hazard ratios obtained from the continuous variable analysis, sex- and age-specific alcohol consumption prevalence data from the 2020-2021 National Health Survey(1), and sex- and age-specific national cancer incidence estimates for 2024 from the Australian Institute of Health and Welfare(2) were used to calculate the cumulative absolute risk of cancer in Australians from age 25 to 85 years in 2024 by alcohol consumption status. These datasets were the most recent years available. This method has been used in previous studies examining alcohol consumption and smoking status and cumulative risk of health outcomes in the 45 and Up Study(3-5). Cumulative risk was calculated by sex for three drinking groups: Persons consuming 0 to <1 drink per week (never-drinkers, former drinkers and occasional drinkers who consumed <1 drink per week), persons consuming ≥1 to ≤10 drinks per week, and persons consuming >10 drinks per week. The upper cut-point of 10 drinks per week was chosen to align with the Australian alcohol consumption guideline of ≤10 standard drinks per week to reduce the risk of alcohol-related harm(6). Age groups in five-year increments were used: 25-29 years, 30-34 years … 80-84 years. In each sex and age group, the absolute rates of a specific type of cancer in persons consuming >10 drinks per week (A_>10_), persons consuming 0 to <1 drink per week (A_0_) and persons consuming ≥1 to ≤10 drinks per week (A_≤10_) were given by:

A_>10_ = A / (P_>10_ + P_≤10_ x HR_≤10_ / HR_>10_ + P_0_ / HR_>10_)

A_0_ = A / (P_>10_ x HR_>10_ + P_≤10_ x HR_≤10_ + P_0_)

A_≤10_ = A_0_ x HR_≤10_

Where A = the Australian incidence rate for this cancer type in this sex and age group, P_0_ = the national prevalence of persons consuming 0 to <1 drink per week for this sex and age group, P_≤10_ = the national prevalence of persons consuming ≥1 to ≤10 drinks per week for this sex and age group, P_>10_ = the national prevalence of persons consuming >10 drinks per week for this sex and age group, HR_≤10_ = the hazard ratio for this cancer type in persons consuming ≥1 to ≤10 drinks per week compared to persons consuming 0 to <1 drink per week derived from the continuous variable analysis of weekly alcohol consumption (calculated for the median drinker within this group in the 45 and Up Study: 5 drinks per week for both men and women), and HR_>10_ = the hazard ratio for this cancer type in persons consuming >10 drinks per week compared to persons consuming 0 to <1 drink per week derived from the continuous variable analysis of weekly alcohol consumption (calculated for the median drinker within this group in the 45 and Up Study: 20 drinks per week for men and 14 drinks per week for women). Due to the detection of an interaction between alcohol consumption and sex for liver cancer and alcohol-related cancers combined, sex-specific hazard ratios were used in the calculations for these outcomes, while non-sex-specific hazard ratios were used for other outcomes. Absolute incidence for each cancer type was then calculated for each sex and five-year age group ($i$), by:

1 - exp(-5 $\sum_{i=(25-29)}^{x} A_{i}$)

Where *x* = age 30, 35 … 85 years, and A_i_ = A_0_, A_≤10_ or A_>10_ for each five-year age group. These were then summed to calculate the sex-specific cumulative absolute risk of cancer from age 25 years to age *x* years by drinking status. This was performed for cancers of the upper aerodigestive tract, mouth and pharynx, oesophagus, larynx, colorectum, colon, rectum, liver and breast, and for alcohol-related cancers combined.

In the sensitivity analysis including additional cancer types, cumulative absolute risks for cancers of the pancreas and lung and melanoma were also estimated. As the hazard ratio point estimate for stomach cancer was less than 1, it was not possible to calculate estimates for stomach cancer.

It should be noted that the continuous variable analysis excluded non-drinkers, and that the calculation of cumulative absolute risk in non-drinkers assumed that the increased cancer risk per ten drink increase in weekly alcohol consumption among drinkers can be extrapolated to non-drinkers. An alternative approach would be to include non-drinkers in the continuous variable analysis, however this would potentially increase the level of bias in the absolute risk estimates due to the problem of the ‘sick-quitter effect’ among non-drinkers. This is a vexed problem, and we have decided to exclude non-drinkers from the continuous variable analysis when calculating cumulative absolute risk as we consider these values likely to be less biased.

Some other limitations should also be noted. Firstly, this analysis assumed that the relationship between alcohol consumption and cancer risk does not differ by age, enabling cancer risk attributable to drinking between the ages of 25 and 45 years to be estimated. Also, the level of alcohol consumption of the median participant consuming >10 drinks per week in the 45 and Up Study may be lower than that of the Australian population if the 45 and Up Study is a ‘healthy cohort’. This would result in an underestimation of the level of risk for the >10 drinks per week group. To a lesser extent, differences in the median level of alcohol consumption between participants in the 45 and Up Study and the Australian population may also bias estimates for the ≥1 to ≤10 drinks per week group.

*Population attributable fractions*

The hazard ratios obtained from the continuous variable analysis, sex- and age-specific alcohol consumption prevalence data from the 2011-2012 Australian Health Survey(7, 8), and sex- and age-specific national cancer incidence estimates for 2024 from the Australian Institute of Health and Welfare(2) were used to calculate sex-specific population attributable fractions (PAF) for alcohol consumption and cancer in Australia in 2024.

The 2011-12 Australian Health Survey data was categorised into 14 drinking groups by average alcohol consumption per day: None; >0 to <1 g; ≥1 to <2 g; ≥2 to <5; ≥5 to <10 g; ≥10 to <15 g; ≥15 to <20 g; ≥20 to <25 g; ≥25 to <30 g; ≥30 to <35 g; ≥35 to <45 g; ≥45 to <55 g; ≥55 to <65 g; ≥65 g. When former drinking were accounted for in the calculation there were 15 drinking groups, with the drinking group ‘None’ divided into ‘Never drinking’ and ‘Former drinking’. The hazard ratio for each drinking group was calculated using the sex-specific median level of intake within each category (except for former drinking, where relative risks from Rumgay et al., (2021)(9) were used due to estimates for former drinking not being available from the 45 and Up Study). Cancer diagnoses in each age group in 2024 were attributed to the alcohol consumption of the age group 10 years younger in 2011-2012 (i.e., the analysis assumed a lag time of approximately 10 years between alcohol consumption and cancer risk. This is because the lag time between alcohol consumption and cancer risk is thought to be at least 10 years(10)). For each sex, age and level of drinking group, the PAF was given by:

$$PAF=\frac{F(HR-1)}{1+F\left( HR-1 \right)}$$

Where F = the proportion of the sex and age group in the drinking group, and HR = the hazard ratio corresponding to the median level of alcohol consumption in grams per day of the drinking group. For this calculation it was assumed that each drink per week in the 45 and Up Study was equivalent to 10 grams of alcohol. Due to the detection of an interaction between alcohol consumption and sex for liver cancer, sex-specific hazard ratios were used in the calculations for liver cancer, while non-sex-specific hazard ratios were used for other cancer types. After summing the PAFs for all drinking groups in a sex and age group, the summed PAF was multiplied by the number of cases for the cancer type in question. This gave the number of excess cancer cases attributable to alcohol consumption, compared to a theoretical minimum risk exposure of no alcohol consumption. The excess cancer cases were then summed for all sex and age groups to obtain the overall PAF. This was performed for cancers of the upper aerodigestive tract, mouth and pharynx, oesophagus, larynx, colorectum, colon, rectum, liver and breast. PAFs for alcohol-related cancers combined and all cancers combined were calculated by summing the number of cancers attributable to alcohol consumption from the calculations for the individual cancer types.

To calculate the potential impact fraction of a reduction in population alcohol consumption, all persons who consumed >10 drinks per week were assigned to exactly 10 drinks per week in the PAF calculation (the upper limit of the Australian weekly alcohol guideline(6)). The potential impact fraction was the difference in the number of cancer cases attributable to alcohol consumption between this analysis and the main analysis.

In the sensitivity analysis including additional cancer types, PAFs for cancers of the stomach, pancreas and lung and melanoma were also estimated. As the hazard ratio point estimate for stomach cancer was less than 1, it was not possible to calculate estimates for current alcohol consumption for stomach cancer. In addition, as relative risks for former drinking for lung cancer and melanoma were not reported in Rumgay et al., (2021)(9), it was not possible to calculate estimates for former drinking for lung cancer and melanoma.

It should be noted that the continuous variable analysis excluded non-drinkers, and that the calculation of cumulative absolute risk in non-drinkers assumed that the increased cancer risk per ten drink increase in weekly alcohol consumption among drinkers can be extrapolated to non-drinkers. An alternative approach would be to include non-drinkers in the continuous variable analysis, however this would potentially increase the level of bias in the absolute risk estimates due to the problem of the ‘sick-quitter effect’ among non-drinkers. This is a vexed problem, and we have decided to exclude non-drinkers from the continuous variable analysis when calculating population attributable fractions as we consider these values likely to be less biased.

**Additional results**

*Proportional hazards assumption violations*

Statistically significant violations of the proportional hazards assumption were detected in models for colon, liver, breast and stomach cancer, melanoma, alcohol-related cancers combined and all cancers combined. For some covariates, plotting the log-log graphs did not reveal clear proportional hazards violations (Supplementary Table 10). Stratified Cox models for the remaining covariates with a statistically significant proportional hazards assumption violation were calculated (results not shown). For the model with a statistically significant proportional hazards assumption violation for alcohol consumption (colon cancer), Cox models stratified by age are presented in Supplementary Table 11. Models without stratification were reported in the manuscript for ease of interpretation.

**Supplementary Table 1. Covariates included in models by cancer type in the 45 and Up Study (2005-2019).**

| **Cancer type (ICD-10 code)** | **Remoteness, education, household income, health insurance status, partner status, country of birth, smoking status and intensity, body mass index, physical activity** | **Sex** | **Fruit and vegetable intake** | **Fibre, red meat and processed meat intake** | **Time spent outdoors, skin tone** | **Parity and age at first birth, breastfeeding duration, menopausal status** | **HC use** | **MHT use** | **Aspirin use** | **Bowel, breast and prostate screening history** |
| --- | --- | --- | --- | --- | --- | --- | --- | --- | --- | --- |
| Upper aerodigestive tract (C00-15;32) | Yes | Yes | Yes | - | - | - | - | - | Yes | - |
| - Mouth and pharynx (C00-14) | Yes | Yes | Yes | - | - | - | - | - | - | - |
| - Oesophagus (C15) | Yes | Yes | Yes | - | - | - | - | - | Yes | - |
| - Larynx (C32) | Yes | Yes | Yes | - | - | - | - | - | - | - |
| Colorectum (C18-20) | Yes | Yes | Yes | Yes | - | - | - | Yes | Yes | Bowel |
| - Colon (C18) | Yes | Yes | Yes | Yes | - | - | - | Yes | Yes | Bowel |
| - Rectum (C19-20) | Yes | Yes | Yes | Yes | - | - | - | Yes | Yes | Bowel |
| Liver (C22) | Yes | Yes | - | - | - | - | Yes | - | - | - |
| Breast (C50^c^) | Yes | - | - | - | - | Yes | Yes | Yes | - | Breast |
| **Alcohol-related cancers combined (C00-15;18-20;22;32;50^c^)** | Yes | Yes | Yes | Yes | - | Yes | Yes | Yes | Yes | Bowel/Breast |
| **All cancers combined (C00-97;D45-46;47.1;47.3-47.4)** | Yes | Yes | Yes | Yes | Yes | Yes | Yes | Yes | Yes | All three |
| *Additional cancer types* |  |  |  |  |  |  |  |  |  |  |
| Stomach (C16) | Yes | Yes | Yes | - | - | - | - | - | - | - |
| Pancreas (C25) | Yes | Yes | - | - | - | - | - | - | - | - |
| Lung (C33-34) | Yes | Yes | Yes | - | - | - | - | - | - | - |
| Melanoma (C43) | Yes | Yes | - | - | Yes | - | - | - | - | - |

Variables obtained from the 45 and Up Study baseline questionnaire (<https://www.saxinstitute.org.au/solutions/45-and-up-study/use-the-45-and-up-study/data-and-technical-information/>). All models adjusted for age through use of age as the underlying time variable. ^a^Breast cancer in women only. BMI, Body Mass Index. HC, Hormonal Contraceptive. ICD-10, International Classification of Diseases, version 10. MHT, Menopausal Hormone Therapy.

**Supplementary Table 2. Categories used and missing data in model covariates.**

| **Covariate** | **Categories** | **Missing data (%)** |
| --- | --- | --- |
| Sex | Man; Woman. | 0.0 |
| Remoteness | Major city; Inner regional; Outer regional; Remote or very remote. | 1.9 |
| Highest level of education | No school certificate or other qualifications; School or intermediate certificate; Higher school or leaving certificate; Trade/apprenticeship; Certificate/diploma; University degree or higher. | 1.5 |
| Annual household income^a^ | <$20,000; ≥$20,000 to <$40,000; ≥$40,000 to <$70,000; ≥$70,000. | 21.0 |
| Health insurance status | Private health insurance - with extras; Private health insurance - without extras; Department of Veterans’ Affairs white or gold card; Health care concession card; None of these. | 1.8 |
| Partner status | Married/living with partner; Not married/living with partner. | 0.6 |
| Country of birth | Australia; Canada/Ireland/New Zealand/United Kingdom/United States of America; Other country. | 0.8 |
| Smoking status and intensity | Never-smoked; Formerly smoked (≤15 cigarettes/day); Formerly smoked (>15 cigarettes/day); Formerly smoked (missing cigarettes/day); Currently smoke (≤15 cigarettes/day); Currently smoke (>15 cigarettes/day); Currently smoke (missing cigarettes/day). | 0.3 |
| Body mass index | Underweight (BMI <18.5 kgm^-2^); Healthy weight (BMI ≥18.5 to <25 kgm^-2^); Overweight (BMI ≥25 to <30 kgm^-2^); Obese (BMI ≥30 kgm^-2^). | 7.3 |
| Physical activity^b^ | Inactive (0 minutes/week); Insufficient (>0 to <150 minutes/week); Sufficient (≥150 to <300 minutes/week); High (≥300 minutes/week). | 2.7 |
| Fruit consumption^c^ | <1 serve/day; ≥1 to <2 serves/day; ≥2 serves/day. | 2.4 |
| Vegetable consumption | <3 serves/day; ≥3 to <5 serves/day; ≥5 serves/day. | 2.4 |
| Fibre intake^d^ | <7 serves/week; ≥7 to <14 serves/week; ≥14 to <21 serves/week; ≥21 serves/week. | 9.1 |
| Red meat consumption | 0 times/week; >0 to ≤2 times/week; >2 to ≤5 times/week; >5 times/week. | 2.0 |
| Processed meat consumption | 0 times/week; >0 to ≤1 times/week; >1 to ≤2 times/week; >2 times/week. | 2.0 |
| Time spent outdoors | <2 hours/day; ≥2 to <4 hours/day; ≥4 to <6 hours/day; ≥6 hours/day. | 4.0 |
| Skin tone | Very fair or fair; Light olive or dark olive; Brown or black. | 1.2 |
| Parity and age at first birth | No children; 1 child (<25 years); 1 child (≥25 years); 2 children (<25 years); 2 children (≥25 years); ≥3 children (<25 years); ≥3 children (≥25 years); Male indicator. | 4.3^e^ |
| Breastfeeding duration | Never breastfed; >0 to ≤12 months; >12 to ≤24 months; >24 months; Male indicator. | 1.8^e^ |
| Menopausal status | Pre-menopausal; Irregular periods; Post-menopausal; Male indicator. | 16.3^e^ |
| Hormonal contraceptive use | Never used; Ever used; Male indicator. | 1.7^e^ |
| Menopausal hormone therapy use | Never used; Formerly used; Current user; Male indicator. | 2.3^e^ |
| Aspirin use | No; Yes. | <0.1 |
| Bowel screening history | Not in the last 10 years; Yes, ≥2 to ≤10 years ago; Yes, <2 years ago. | 4.4 |
| Breast screening history | Not in the last 10 years; Yes, ≥2 to ≤10 years ago; Yes, <2 years ago; Male indicator. | 5.4^e^ |
| Prostate screening history | Never; Yes, 1-3 times; Yes, >3 times; Yes, times missing; Female indicator. | 8.0^f^ |

Variables derived from the 45 and Up Study baseline questionnaire (<https://www.saxinstitute.org.au/solutions/45-and-up-study/use-the-45-and-up-study/data-and-technical-information/>). All categorical covariates except sex had a missing indicator category. ^a^Pre-tax annual household income from all sources in Australian dollars. ^b^Each minute of walking or moderate physical activity counted as 1 minute, and each minute of vigorous physical activity counted as 2 minutes, according to the Australian physical activity guidelines(11). ^c^Excludes fruit juice. ^d^Serves of breakfast cereal and brown or wholemeal bread. ^e^Missing data percentage among women. ^f^Missing data percentage among men. BMI, Body Mass Index.

**Supplementary Table 3. Additional analyses for hazard ratios (HR) and 95% confidence intervals (CI) of cancer risk per ten drink increase in weekly alcohol consumption among drinkers in the 45 and Up Study (2005-2019).**

|  | **Main analysis** | |  | **Exclusion of the first year of follow-up** | |  | **Using the mean number of drinks per week of each category of intake reported at baseline rather than at 5-year follow-up** | |  | **Using the actual number of drinks per week reported by participants rather than the mean of each category of intake** | |  | **Using a dose-response increment of 1 drink per week rather than 10 drinks per week** | |
| --- | --- | --- | --- | --- | --- | --- | --- | --- | --- | --- | --- | --- | --- | --- |
| **Cancer type (ICD-10 code)** | **n cases** | **HR (95% CI)** |  | **n cases** | **HR (95% CI)** |  | **n cases** | **HR (95% CI)** |  | **n cases** | **HR (95% CI)** |  | **n cases** | **HR (95% CI)** |
| Upper aerodigestive tract (C00-15;32) | 972 | 1.27 (1.18-1.37) |  | 899 | 1.28 (1.18-1.38) |  | 972 | 1.19 (1.13-1.26) |  | 972 | 1.18 (1.13-1.23) |  | 972 | 1.024 (1.017-1.032) |
| - Mouth and pharynx (C00-14) | 621 | 1.27 (1.16-1.39) |  | 577 | 1.29 (1.17-1.42) |  | 621 | 1.19 (1.11-1.27) |  | 621 | 1.17 (1.11-1.24) |  | 621 | 1.024 (1.015-1.034) |
| - Oesophagus (C15) | 248 | 1.29 (1.11-1.49) |  | 226 | 1.33 (1.14-1.55) |  | 248 | 1.21 (1.09-1.34) |  | 248 | 1.20 (1.10-1.31) |  | 248 | 1.026 (1.011-1.041) |
| - Larynx (C32) | 108 | 1.22 (0.99-1.50) |  | 100 | 1.11 (0.89-1.39) |  | 108 | 1.17 (1.01-1.35) |  | 108 | 1.16 (1.03-1.31) |  | 108 | 1.022 (0.999-1.042) |
| Colorectum (C18-20) | 2,889 | 1.16 (1.11-1.22) |  | 2,636 | 1.17 (1.11-1.23) |  | 2,889 | 1.12 (1.08-1.16) |  | 2,889 | 1.10 (1.07-1.14) |  | 2,889 | 1.015 (1.010-1.020) |
| - Colon (C18) | 2,012 | 1.18 (1.11-1.25) |  | 1,851 | 1.17 (1.10-1.25) |  | 2,012 | 1.13 (1.08-1.18) |  | 2,012 | 1.12 (1.07-1.16) |  | 2,012 | 1.017 (1.011-1.023) |
| - Rectum (C19-20) | 914 | 1.15 (1.06-1.25) |  | 816 | 1.17 (1.07-1.28) |  | 914 | 1.10 (1.04-1.17) |  | 914 | 1.09 (1.03-1.15) |  | 914 | 1.014 (1.006-1.023) |
| Liver (C22) | 258 | 1.46 (1.28-1.68) |  | 246 | 1.46 (1.27-1.68) |  | 258 | 1.32 (1.20-1.45) |  | 258 | 1.23 (1.14-1.32) |  | 258 | 1.039 (1.025-1.053) |
| Breast (C50^a^) | 2,891 | 1.18 (1.09-1.28) |  | 2,653 | 1.16 (1.07-1.27) |  | 2,891 | 1.12 (1.06-1.19) |  | 2,891 | 1.12 (1.06-1.18) |  | 2,891 | 1.017 (1.009-1.025) |
| **Alcohol-related cancers combined (C00-15;18-20;22;32;50^a^)** | 6,923 | 1.19 (1.15-1.23) |  | 6,356 | 1.19 (1.15-1.23) |  | 6,923 | 1.14 (1.11-1.17) |  | 6,923 | 1.13 (1.11-1.16) |  | 6,923 | 1.018 (1.014-1.021) |
| **All cancers combined (C00-97;D45-46;47.1;47.3-47.5)** | 24,133 | 1.05 (1.03-1.07) |  | 22,198 | 1.05 (1.03-1.07) |  | 24,133 | 1.03 (1.02-1.05) |  | 24,133 | 1.03 (1.02-1.04) |  | 24,133 | 1.005 (1.003-1.006) |

Linear trend in categories calculated among drinkers only, where participants within each category of alcohol consumption at baseline were assigned the mean level of alcohol consumption they reported at first wave follow-up (median 5.3 years after baseline). Models were adjusted for cancer-specific covariates as listed in Supplementary Table 1. Cancer cases do not sum to totals as some participants were diagnosed with two or more primary cancers. ^a^Breast cancer in women only. ICD-10, International Classification of Diseases, version 10.

**Supplementary Table 4. Interaction tests and stratifications for hazard ratios (HR) and 95% confidence intervals (CI) of cancer risk per ten drink increase in weekly alcohol consumption among drinkers in the 45 and Up Study (2005-2019).**

| **Cancer type (ICD-10 code)** | **n cases** | **Main analysis** | **Men** | **Women** | ***p*_interaction (sex)_** | **Never smoked** | **Formerly smoked** | **Currently smoke** | ***p*_interaction (smoking)_** | **Born in Australia** | **Born in other countries** | ***p*_interaction (COB)_** | ***p*_interaction (SES)_** |
| --- | --- | --- | --- | --- | --- | --- | --- | --- | --- | --- | --- | --- | --- |
| Upper aerodigestive tract (C00-15;32) | 972 | 1.27 (1.18-1.37) | - | - | 0.83 | - | - | - | 0.14 | - | - | 0.15 | 0.64 |
| - Mouth and pharynx (C00-14) | 621 | 1.27 (1.16-1.39) | - | - | 0.81 | - | - | - | 0.08 | - | - | 0.56 | 0.81 |
| - Oesophagus (C15) | 248 | 1.29 (1.11-1.49) | - | - | 0.96 | 0.71 (0.45-1.11) | 1.24 (1.02-1.50) | 1.92 (1.40-2.65) | 0.006 | - | - | 0.16 | 0.17 |
| - Larynx (C32) | 108 | 1.22 (0.99-1.50) | - | - | 0.93 | - | - | - | 0.53 | - | - | 0.20 | 0.28 |
| Colorectum (C18-20) | 2,889 | 1.16 (1.11-1.22) | - | - | 0.16 | - | - | - | 0.46 | - | - | 0.12 | 0.79 |
| - Colon (C18) | 2,012 | 1.18 (1.11-1.25) | - | - | 0.09 | - | - | - | 0.35 | 1.16 (1.09-1.24) | 1.31 (1.15-1.48) | 0.047 | 0.98 |
| - Rectum (C19-20) | 914 | 1.15 (1.06-1.25) | - | - | 0.99 | - | - | - | 0.52 | - | - | 0.89 | 0.56 |
| Liver (C22) | 258 | 1.46 (1.28-1.68) | 1.39 (1.20-1.61) | 2.03 (1.29-3.19) | 0.04 | - | - | - | 0.91 | - | - | 0.20 | 0.09 |
| Breast (C50^a^) | 2,891 | 1.18 (1.09-1.28) | - | - | - | - | - | - | 0.35 | - | - | 0.58 | 0.38 |
| **Alcohol-related cancers combined (^b^)** | 6,923 | 1.19 (1.15-1.23) | 1.21 (1.16-1.26) | 1.17 (1.09-1.25) | 0.01 | 1.16 (1.09-1.23) | 1.17 (1.12-1.23) | 1.27 (1.16-1.39) | 0.009 | - | - | 0.39 | 0.80 |
| **All cancers combined (^c^)** | 24,133 | 1.05 (1.03-1.07) | - | - | 0.62 | - | - | - | 0.11 | - | - | 0.15 | 0.81 |

Linear trend in categories calculated among drinkers only, where participants within each category of alcohol consumption at baseline were assigned the mean level of alcohol consumption they reported at first wave follow-up (median 5.3 years after baseline). Models were adjusted for cancer-specific covariates as listed in Supplementary Table 1. Cancer cases do not sum to totals as some participants were diagnosed with two or more primary cancers. ^a^Breast cancer in women only. ^b^C00-15;18-20;22;32;50, including breast cancer in women only. ^c^C00-97;D45-46;47.1;47.3-47.5. COB, Country Of Birth. ICD-10, International Classification of Diseases, version 10. SES, Socio-Economic Status.

**Supplementary Table 5. Hazard ratios (HR) and 95% confidence intervals (CI) of cancer risk by pattern of drinking among participants consuming ≥4 drinks per week in the 45 and Up Study (2005-2019).**

|  |  | **4-7 drinking days per week** | | |  | **1-3 drinking days per week** | | |  |
| --- | --- | --- | --- | --- | --- | --- | --- | --- | --- |
| **Cancer type (ICD-10 code)** | **n cases** | **≥4 to ≤7 drinks** | **>7 to ≤10 drinks** | **>10 drinks** |  | **≥4 to ≤7 drinks** | **>7 to ≤10 drinks** | **>10 drinks** | ***p*_interaction_^a^** |
| Upper aerodigestive tract (C00-15;32) | 790 | 1.00 | 1.21 (0.92-1.58) | 1.38 (1.11-1.71) |  | 0.91 (0.68-1.22) | 1.46 (1.02-2.08) | 1.22 (0.81-1.85) | 0.40 |
| Colorectum (C18-20) | 2,282 | 1.00 | 1.02 (0.88-1.18) | 1.31 (1.16-1.47) |  | 1.01 (0.87-1.18) | 1.20 (0.96-1.51) | 1.27 (0.98-1.66) | 0.46 |
| - Colon (C18) | 1,591 | 1.00 | 1.00 (0.84-1.20) | 1.29 (1.13-1.48) |  | 1.05 (0.88-1.26) | 1.26 (0.96-1.66) | 1.50 (1.10-2.05) | 0.56 |
| - Rectum (C19-20) | 719 | 1.00 | 1.08 (0.83-1.42) | 1.39 (1.12-1.73) |  | 0.95 (0.72-1.26) | 1.13 (0.76-1.68) | 0.93 (0.58-1.55) | 0.36 |
| Breast (C50^b^) | 2,100 | 1.00 | 1.07 (0.94-1.22) | 1.18 (1.06-1.33) |  | 0.98 (0.86-1.13) | 1.18 (0.93-1.49) | 1.31 (0.91-1.89) | 0.67 |
| **Alcohol-related cancers combined (C00-15;18-20;22;32;50^b^)** | 5,307 | 1.00 | 1.08 (0.98-1.18) | 1.26 (1.17-1.36) |  | 1.00 (0.91-1.10) | 1.21 (1.04-1.40) | 1.27 (1.06-1.52) | 0.43 |
| **All cancers combined (C00-97;D45-46;47.1;47.3-47.5)** | 18,929 | 1.00 | 1.06 (1.01-1.11) | 1.13 (1.09-1.18) |  | 1.09 (1.04-1.15) | 1.18 (1.09-1.27) | 1.14 (1.04-1.25) | 0.19 |

Models were adjusted for cancer-specific covariates as listed in Supplementary Table 1. Cancer cases do not sum to totals as some participants were diagnosed with two or more primary cancers. ^a^Test of interaction between days per week and drinks per week. ^b^Breast cancer in women only. ICD-10, International Classification of Diseases, version 10.

**Supplementary Table 6. Hazard ratios (HR) and 95% confidence intervals (CI) of cancer risk by pattern of drinking among participants consuming ≥4 drinks per week in the 45 and Up Study (2005-2019), using categorisation based on the former Australian alcohol consumption guidelines in a previous analysis of the 45 and Up Study.**

|  |  | **4-7 drinking days per week** | | |  | **1-3 drinking days per week** | | |  |
| --- | --- | --- | --- | --- | --- | --- | --- | --- | --- |
| **Cancer type (ICD-10 code)** | **n cases** | **≥4 to <7 drinks** | **≥7 to <14 drinks** | **≥14 drinks** |  | **≥4 to <7 drinks** | **≥7 to <14 drinks** | **≥14 drinks** | ***p*_interaction_^a^** |
| Upper aerodigestive tract (C00-15;32) | 790 | 1.00 | 0.98 (0.74-1.30) | 1.25 (0.96-1.64) |  | 0.81 (0.58-1.13) | 1.24 (0.88-1.76) | 0.99 (0.59-1.69) | 0.08 |
| Colorectum (C18-20) | 2,282 | 1.00 | 0.95 (0.82-1.10) | 1.24 (1.07-1.43) |  | 0.96 (0.81-1.14) | 1.09 (0.88-1.34) | 1.17 (0.83-1.63) | 0.35 |
| - Colon (C18) | 1,591 | 1.00 | 0.92 (0.77-1.09) | 1.19 (1.00-1.40) |  | 0.99 (0.81-1.21) | 1.08 (0.84-1.40) | 1.40 (0.95-2.06) | 0.46 |
| - Rectum (C19-20) | 719 | 1.00 | 1.06 (0.80-1.40) | 1.41 (1.07-1.85) |  | 0.93 (0.67-1.29) | 1.14 (0.78-1.67) | 0.77 (0.39-1.50) | 0.16 |
| Breast (C50^b^) | 2,100 | 1.00 | 0.97 (0.85-1.10) | 1.11 (0.97-1.28) |  | 0.92 (0.79-1.07) | 1.03 (0.83-1.28) | 1.67 (1.07-2.61) | 0.09 |
| **Alcohol-related cancers combined (C00-15;18-20;22;32;50^b^)** | 5,307 | 1.00 | 0.95 (0.87-1.04) | 1.17 (1.07-1.28) |  | 0.92 (0.83-1.03) | 1.05 (0.92-1.21) | 1.26 (1.01-1.57) | 0.07 |
| **All cancers combined (C00-97;D45-46;47.1;47.3-47.5)** | 18,929 | 1.00 | 1.01 (0.96-1.07) | 1.12 (1.07-1.18) |  | 1.07 (1.00-1.13) | 1.15 (1.07-1.23) | 1.12 (1.00-1.26) | 0.10 |

Models were adjusted for cancer-specific covariates as listed in Supplementary Table 1. Categorisation used based on the former Australian alcohol consumption guidelines in a previous analysis of the 45 and Up Study(4). Cancer cases do not sum to totals as some participants were diagnosed with two or more primary cancers. ^a^Test of interaction between days per week and drinks per week. ^b^Breast cancer in women only. ICD-10, International Classification of Diseases, version 10.

**Supplementary Table 7. Cumulative absolute risk (%) of cancer diagnosis from age 25 to 85 years in Australia in 2024 by sex and level of alcohol consumption using hazard ratios from the 45 and Up Study (2005-2019).**

| **Sex and age** | **Upper aerodigestive tract (drinks/week)** | | |  | **Mouth and pharynx (drinks/week)** | | |  | **Oesophagus**  **(drinks/week)** | | |  | **Larynx**  **(drinks/week)** | | |  | **Colorectum**  **(drinks/week)** | | |
| --- | --- | --- | --- | --- | --- | --- | --- | --- | --- | --- | --- | --- | --- | --- | --- | --- | --- | --- | --- |
|  | **0 to <1** | **≥1 to ≤10** | **>10** |  | **0 to <1** | **≥1 to ≤10** | **>10** |  | **0 to <1** | **≥1 to ≤10** | **>10** |  | **0 to <1** | **≥1 to ≤10** | **>10** |  | **0 to <1** | **≥1 to ≤10** | **>10** |
| **Men** |  |  |  |  |  |  |  |  |  |  |  |  |  |  |  |  |  |  |  |
| 25 years | 0.00 | 0.00 | 0.00 |  | 0.00 | 0.00 | 0.00 |  | 0.00 | 0.00 | 0.00 |  | 0.00 | 0.00 | 0.00 |  | 0.00 | 0.00 | 0.00 |
| 30 years | 0.01 | 0.01 | 0.01 |  | 0.00 | 0.01 | 0.01 |  | 0.00 | 0.00 | 0.00 |  | 0.00 | 0.00 | 0.00 |  | 0.03 | 0.03 | 0.04 |
| 35 years | 0.02 | 0.02 | 0.02 |  | 0.01 | 0.01 | 0.02 |  | 0.00 | 0.00 | 0.00 |  | 0.00 | 0.00 | 0.00 |  | 0.09 | 0.10 | 0.13 |
| 40 years | 0.04 | 0.04 | 0.06 |  | 0.03 | 0.04 | 0.06 |  | 0.00 | 0.00 | 0.01 |  | 0.00 | 0.00 | 0.00 |  | 0.18 | 0.19 | 0.24 |
| 45 years | 0.09 | 0.10 | 0.14 |  | 0.08 | 0.09 | 0.13 |  | 0.01 | 0.01 | 0.01 |  | 0.00 | 0.00 | 0.00 |  | 0.28 | 0.30 | 0.38 |
| 50 years | 0.19 | 0.21 | 0.30 |  | 0.17 | 0.19 | 0.27 |  | 0.02 | 0.02 | 0.03 |  | 0.00 | 0.00 | 0.01 |  | 0.44 | 0.48 | 0.60 |
| 55 years | 0.38 | 0.42 | 0.61 |  | 0.31 | 0.35 | 0.50 |  | 0.04 | 0.05 | 0.07 |  | 0.02 | 0.02 | 0.03 |  | 0.75 | 0.81 | 1.02 |
| 60 years | 0.68 | 0.76 | 1.10 |  | 0.54 | 0.60 | 0.86 |  | 0.10 | 0.11 | 0.17 |  | 0.04 | 0.05 | 0.06 |  | 1.05 | 1.14 | 1.43 |
| 65 years | 1.13 | 1.27 | 1.82 |  | 0.86 | 0.97 | 1.39 |  | 0.19 | 0.22 | 0.32 |  | 0.08 | 0.09 | 0.12 |  | 1.61 | 1.74 | 2.19 |
| 70 years | 1.61 | 1.82 | 2.60 |  | 1.18 | 1.33 | 1.90 |  | 0.30 | 0.34 | 0.50 |  | 0.13 | 0.14 | 0.19 |  | 2.26 | 2.43 | 3.06 |
| 75 years | 2.19 | 2.47 | 3.54 |  | 1.50 | 1.69 | 2.42 |  | 0.49 | 0.56 | 0.82 |  | 0.20 | 0.23 | 0.30 |  | 3.17 | 3.42 | 4.30 |
| 80 years | 2.77 | 3.13 | 4.49 |  | 1.83 | 2.06 | 2.95 |  | 0.68 | 0.77 | 1.12 |  | 0.27 | 0.30 | 0.41 |  | 4.19 | 4.52 | 5.68 |
| 85 years | 3.40 | 3.83 | 5.50 |  | 2.14 | 2.41 | 3.45 |  | 0.92 | 1.04 | 1.52 |  | 0.36 | 0.39 | 0.53 |  | 5.84 | 6.30 | 7.92 |
| **Women** |  |  |  |  |  |  |  |  |  |  |  |  |  |  |  |  |  |  |  |
| 25 years | 0.00 | 0.00 | 0.00 |  | 0.00 | 0.00 | 0.00 |  | 0.00 | 0.00 | 0.00 |  | 0.00 | 0.00 | 0.00 |  | 0.00 | 0.00 | 0.00 |
| 30 years | 0.01 | 0.01 | 0.01 |  | 0.01 | 0.01 | 0.01 |  | 0.00 | 0.00 | 0.00 |  | 0.00 | 0.00 | 0.00 |  | 0.03 | 0.04 | 0.04 |
| 35 years | 0.02 | 0.02 | 0.02 |  | 0.01 | 0.02 | 0.02 |  | 0.00 | 0.00 | 0.00 |  | 0.00 | 0.00 | 0.00 |  | 0.10 | 0.11 | 0.13 |
| 40 years | 0.03 | 0.03 | 0.04 |  | 0.03 | 0.03 | 0.04 |  | 0.00 | 0.00 | 0.00 |  | 0.00 | 0.00 | 0.00 |  | 0.20 | 0.22 | 0.25 |
| 45 years | 0.05 | 0.06 | 0.07 |  | 0.04 | 0.05 | 0.06 |  | 0.00 | 0.00 | 0.00 |  | 0.00 | 0.00 | 0.00 |  | 0.33 | 0.36 | 0.41 |
| 50 years | 0.08 | 0.09 | 0.12 |  | 0.07 | 0.08 | 0.10 |  | 0.01 | 0.01 | 0.01 |  | 0.00 | 0.00 | 0.00 |  | 0.48 | 0.52 | 0.60 |
| 55 years | 0.15 | 0.17 | 0.22 |  | 0.13 | 0.15 | 0.19 |  | 0.02 | 0.02 | 0.02 |  | 0.01 | 0.01 | 0.01 |  | 0.74 | 0.79 | 0.91 |
| 60 years | 0.26 | 0.29 | 0.36 |  | 0.22 | 0.24 | 0.30 |  | 0.03 | 0.04 | 0.05 |  | 0.01 | 0.01 | 0.01 |  | 0.96 | 1.04 | 1.19 |
| 65 years | 0.40 | 0.45 | 0.56 |  | 0.31 | 0.35 | 0.44 |  | 0.06 | 0.07 | 0.09 |  | 0.02 | 0.02 | 0.02 |  | 1.33 | 1.43 | 1.65 |
| 70 years | 0.54 | 0.60 | 0.75 |  | 0.41 | 0.46 | 0.57 |  | 0.10 | 0.11 | 0.14 |  | 0.03 | 0.03 | 0.04 |  | 1.77 | 1.91 | 2.19 |
| 75 years | 0.72 | 0.81 | 1.01 |  | 0.53 | 0.60 | 0.74 |  | 0.16 | 0.18 | 0.22 |  | 0.03 | 0.04 | 0.04 |  | 2.46 | 2.66 | 3.05 |
| 80 years | 0.94 | 1.06 | 1.31 |  | 0.67 | 0.75 | 0.94 |  | 0.23 | 0.26 | 0.32 |  | 0.04 | 0.05 | 0.06 |  | 3.40 | 3.67 | 4.21 |
| 85 years | 1.18 | 1.33 | 1.66 |  | 0.83 | 0.93 | 1.16 |  | 0.31 | 0.35 | 0.43 |  | 0.05 | 0.06 | 0.07 |  | 4.83 | 5.21 | 5.98 |

Calculations based on the results of the continuous variable analysis among 45 and Up Study participants consuming ≥1 drink per week. Three categories of drinking were used: 0 to <1 drink per week (never-drinkers, former drinkers and occasional drinkers who consumed <1 drink per week), ≥1 to ≤10 drinks per week (median 5 drinks in men and women), and >10 drinks per week (median 20 drinks in men and 14 drinks in women). The sums of cumulative risks across individual cancer types may differ from the corresponding risk for combined outcomes (upper aerodigestive tract, colorectal, and alcohol-related cancers combined) because: i) Some Australians were diagnosed with two or more different alcohol-related cancers while the risk of cancer in the 45 and Up Study represents the risk of first diagnosis of alcohol-related cancer only; ii) The hazard ratios used for liver cancer and alcohol-related cancers combined were sex-specific due to the detection of an interaction between alcohol consumption and sex for these outcomes, while the hazard ratios used for other cancer types were not sex-specific.

**Supplementary Table 7. (Continued).**

| **Sex and age** | **Colon**  **(drinks/week)** | | |  | **Rectum**  **(drinks/week)** | | |  | **Liver**  **(drinks/week)** | | |  | **Breast**  **(drinks/week)** | | |  | **Alcohol-related combined**  **(drinks/week)** | | |
| --- | --- | --- | --- | --- | --- | --- | --- | --- | --- | --- | --- | --- | --- | --- | --- | --- | --- | --- | --- |
|  | **0 to <1** | **≥1 to ≤10** | **>10** |  | **0 to <1** | **≥1 to ≤10** | **>10** |  | **0 to <1** | **≥1 to ≤10** | **>10** |  | **0 to <1** | **≥1 to ≤10** | **>10** |  | **0 to <1** | **≥1 to ≤10** | **>10** |
| **Men** |  |  |  |  |  |  |  |  |  |  |  |  |  |  |  |  |  |  |  |
| 25 years | 0.00 | 0.00 | 0.00 |  | 0.00 | 0.00 | 0.00 |  | 0.00 | 0.00 | 0.00 |  | - | - | - |  | 0.00 | 0.00 | 0.00 |
| 30 years | 0.02 | 0.02 | 0.03 |  | 0.01 | 0.01 | 0.01 |  | 0.00 | 0.00 | 0.00 |  | - | - | - |  | 0.03 | 0.04 | 0.05 |
| 35 years | 0.06 | 0.06 | 0.08 |  | 0.04 | 0.04 | 0.05 |  | 0.00 | 0.00 | 0.01 |  | - | - | - |  | 0.11 | 0.12 | 0.16 |
| 40 years | 0.10 | 0.11 | 0.14 |  | 0.07 | 0.08 | 0.10 |  | 0.01 | 0.01 | 0.01 |  | - | - | - |  | 0.22 | 0.24 | 0.32 |
| 45 years | 0.16 | 0.18 | 0.23 |  | 0.12 | 0.13 | 0.16 |  | 0.01 | 0.02 | 0.02 |  | - | - | - |  | 0.38 | 0.42 | 0.56 |
| 50 years | 0.26 | 0.28 | 0.36 |  | 0.18 | 0.19 | 0.24 |  | 0.03 | 0.03 | 0.06 |  | - | - | - |  | 0.66 | 0.72 | 0.96 |
| 55 years | 0.43 | 0.46 | 0.60 |  | 0.32 | 0.34 | 0.42 |  | 0.07 | 0.09 | 0.14 |  | - | - | - |  | 1.20 | 1.32 | 1.76 |
| 60 years | 0.59 | 0.65 | 0.83 |  | 0.46 | 0.49 | 0.61 |  | 0.18 | 0.22 | 0.35 |  | - | - | - |  | 1.94 | 2.13 | 2.83 |
| 65 years | 0.93 | 1.02 | 1.30 |  | 0.68 | 0.73 | 0.90 |  | 0.40 | 0.48 | 0.78 |  | - | - | - |  | 3.20 | 3.52 | 4.67 |
| 70 years | 1.31 | 1.42 | 1.83 |  | 0.94 | 1.01 | 1.25 |  | 0.64 | 0.75 | 1.23 |  | - | - | - |  | 4.59 | 5.04 | 6.69 |
| 75 years | 1.93 | 2.09 | 2.69 |  | 1.24 | 1.33 | 1.64 |  | 0.87 | 1.03 | 1.68 |  | - | - | - |  | 6.34 | 6.97 | 9.24 |
| 80 years | 2.68 | 2.91 | 3.74 |  | 1.50 | 1.61 | 1.99 |  | 1.11 | 1.31 | 2.14 |  | - | - | - |  | 8.20 | 9.01 | 11.95 |
| 85 years | 3.91 | 4.24 | 5.44 |  | 1.92 | 2.06 | 2.54 |  | 1.41 | 1.66 | 2.72 |  | - | - | - |  | 10.78 | 11.85 | 15.69 |
| **Women** |  |  |  |  |  |  |  |  |  |  |  |  |  |  |  |  |  |  |  |
| 25 years | 0.00 | 0.00 | 0.00 |  | 0.00 | 0.00 | 0.00 |  | 0.00 | 0.00 | 0.00 |  | 0.00 | 0.00 | 0.00 |  | 0.00 | 0.00 | 0.00 |
| 30 years | 0.03 | 0.03 | 0.04 |  | 0.00 | 0.01 | 0.01 |  | 0.00 | 0.00 | 0.00 |  | 0.05 | 0.05 | 0.06 |  | 0.09 | 0.10 | 0.11 |
| 35 years | 0.08 | 0.09 | 0.10 |  | 0.02 | 0.03 | 0.03 |  | 0.00 | 0.00 | 0.01 |  | 0.20 | 0.21 | 0.25 |  | 0.32 | 0.35 | 0.40 |
| 40 years | 0.14 | 0.16 | 0.18 |  | 0.06 | 0.06 | 0.07 |  | 0.00 | 0.01 | 0.01 |  | 0.48 | 0.52 | 0.60 |  | 0.72 | 0.78 | 0.89 |
| 45 years | 0.23 | 0.25 | 0.29 |  | 0.10 | 0.11 | 0.13 |  | 0.01 | 0.01 | 0.03 |  | 1.09 | 1.18 | 1.37 |  | 1.48 | 1.60 | 1.85 |
| 50 years | 0.32 | 0.34 | 0.40 |  | 0.17 | 0.18 | 0.20 |  | 0.02 | 0.02 | 0.04 |  | 2.07 | 2.25 | 2.61 |  | 2.66 | 2.88 | 3.32 |
| 55 years | 0.47 | 0.51 | 0.59 |  | 0.27 | 0.29 | 0.32 |  | 0.04 | 0.05 | 0.09 |  | 3.33 | 3.62 | 4.20 |  | 4.28 | 4.63 | 5.33 |
| 60 years | 0.60 | 0.65 | 0.76 |  | 0.36 | 0.38 | 0.43 |  | 0.07 | 0.10 | 0.19 |  | 4.60 | 4.99 | 5.79 |  | 5.92 | 6.40 | 7.38 |
| 65 years | 0.84 | 0.92 | 1.07 |  | 0.48 | 0.52 | 0.59 |  | 0.12 | 0.17 | 0.32 |  | 6.08 | 6.61 | 7.66 |  | 7.98 | 8.63 | 9.94 |
| 70 years | 1.15 | 1.25 | 1.45 |  | 0.62 | 0.66 | 0.75 |  | 0.18 | 0.25 | 0.48 |  | 7.97 | 8.66 | 10.04 |  | 10.52 | 11.38 | 13.11 |
| 75 years | 1.69 | 1.84 | 2.13 |  | 0.77 | 0.82 | 0.94 |  | 0.24 | 0.34 | 0.63 |  | 10.11 | 10.98 | 12.73 |  | 13.61 | 14.71 | 16.94 |
| 80 years | 2.48 | 2.69 | 3.12 |  | 0.92 | 0.98 | 1.11 |  | 0.32 | 0.46 | 0.88 |  | 11.70 | 12.71 | 14.73 |  | 16.45 | 17.79 | 20.48 |
| 85 years | 3.67 | 3.99 | 4.64 |  | 1.15 | 1.23 | 1.40 |  | 0.43 | 0.61 | 1.16 |  | 13.55 | 14.71 | 17.05 |  | 20.08 | 21.72 | 24.99 |

**Supplementary Table 8. Potential impact fractions for cancer caused by alcohol consumption in the Australian population in 2024, if all persons who consumed >10 drinks per week had instead consumed exactly 10 drinks per week, derived using risk estimates from the 45 and Up Study (2005-2019).**

| **Cancer type (ICD-10 code) and sex** | **n alcohol-attributable cases^a^**  **/n total cases** | **n alcohol-attributable cases if all persons consumed ≤10 drinks/week** | **n alcohol-attributable cases prevented if all persons consumed ≤10 drinks/week** | **PIF (%)** |
| --- | --- | --- | --- | --- |
| **Men** |  |  |  |  |
| Upper aerodigestive tract (C00-15;32) | 1,572/5,230 | 629 | 943 | 18.0 |
| - Mouth and pharynx (C00-14) | 1,035/3,411 | 413 | 623 | 18.3 |
| - Oesophagus (C15) | 416/1,323 | 167 | 249 | 18.8 |
| - Larynx (C32) | 121/496 | 49 | 71 | 14.4 |
| Colorectum (C18-20) | 1,528/8,205 | 638 | 890 | 10.8 |
| - Colon (C18) | 1,059/5,448 | 442 | 617 | 11.3 |
| - Rectum (C19-20) | 468/2,757 | 196 | 272 | 9.9 |
| Liver (C22) | 977/2,336 | 381 | 596 | 25.5 |
| Breast (C50^b^) | -/- | - | - | - |
| **Alcohol-related cancers combined (C00-15;18-20;22;32;50^b^)** | 4,076/23,976 | 1647 | 2429 | 10.1 |
| **All cancers combined (C00-97;D45-46;47.1;47.3-47.5)** | 4,076/93,504 | 1647 | 2429 | 2.6 |
|  |  |  |  |  |
| **Women** |  |  |  |  |
| Upper aerodigestive tract (C00-15;32) | 236/1,843 | 134 | 101 | 5.5 |
| - Mouth and pharynx (C00-14) | 166/1,304 | 95 | 72 | 5.5 |
| - Oesophagus (C15) | 61/462 | 35 | 26 | 5.7 |
| - Larynx (C32) | 8/77 | 5 | 3 | 4.5 |
| Colorectum (C18-20) | 557/7,337 | 336 | 220 | 3.0 |
| - Colon (C18) | 431/5,539 | 261 | 170 | 3.1 |
| - Rectum (C19-20) | 126/1,798 | 76 | 50 | 2.8 |
| Liver (C22) | 422/872 | 189 | 233 | 26.7 |
| Breast (C50^b^) | 1,837/20,973 | 1087 | 750 | 3.6 |
| **Alcohol-related cancers combined (C00-15;18-20;22;32;50^b^)** | 3,051/38,362 | 1747 | 1305 | 3.4 |
| **All cancers combined (C00-97;D45-46;47.1;47.3-47.5)** | 3,051/75,974 | 1747 | 1305 | 1.7 |
|  |  |  |  |  |
| **Persons** |  |  |  |  |
| Upper aerodigestive tract (C00-15;32) | 1,808/7,073 | 763 | 1045 | 14.8 |
| - Mouth and pharynx (C00-14) | 1,201/4,715 | 507 | 694 | 14.7 |
| - Oesophagus (C15) | 477/1,785 | 202 | 275 | 15.4 |
| - Larynx (C32) | 129/573 | 54 | 75 | 13.1 |
| Colorectum (C18-20) | 2,084/15,542 | 974 | 1110 | 7.1 |
| - Colon (C18) | 1,490/10,987 | 703 | 788 | 7.2 |
| - Rectum (C19-20) | 594/4,555 | 272 | 322 | 7.1 |
| Liver (C22) | 1,399/3,208 | 570 | 829 | 25.8 |
| Breast (C50^b^) | 1,837/20,973 | 1087 | 750 | 3.6 |
| **Alcohol-related cancers combined (C00-15;18-20;22;32;50^b^)** | 7,128/62,338 | 3394 | 3733 | 6.0 |
| **All cancers combined (C00-97;D45-46;47.1;47.3-47.5)** | 7,128/169,478 | 3394 | 3733 | 2.2 |

Hazard ratios used in calculations for the 45 and Up Study were derived from total alcohol consumption as a log-linear variable among participants consuming ≥1 drink per week. For each age group used in the calculation, cancer incidence was attributed to alcohol consumption approximately 10 years earlier in the 2011-2012 Australian Health Survey. ‘n excess cases’ refers to cancer cases attributable to alcohol consumption out of the total number of cases, ‘n cases’. Cancer cases may not sum to totals due to rounding. ^a^n cancer cases attributable to current alcohol consumption. ^b^Breast cancer in women only. ICD-10, International Classification of Diseases, version 10. PIF, Potential Impact Fraction.

**Supplementary Table 9. Hazard ratios (HR) and 95% confidence intervals (CI) of cancer risk by alcohol consumption, with exclusion of the first year of follow-up, in the 45 and Up Study (2005-2019).**

|  |  | **HR drinks per week (95% CI)** | | | | | |  |
| --- | --- | --- | --- | --- | --- | --- | --- | --- |
| **Cancer type (ICD-10 code)** | **n cases** | **0 to <1** | **≥1 to ≤3.5** | **>3.5 to ≤10** | **>10 to ≤20** | **>20 to ≤30** | **>30** | ***p*^a^** |
| Upper aerodigestive tract (C00-15;32) | 1,218 | 0.90 (0.74-1.09) | 1.00 | 0.98 (0.81-1.19) | 1.06 (0.86-1.31) | 1.38 (1.08-1.76) | 2.22 (1.71-2.88) | <0.001 |
| - Mouth and pharynx (C00-14) | 764 | 0.81 (0.64-1.04) | 1.00 | 1.02 (0.81-1.30) | 1.16 (0.89-1.51) | 1.60 (1.18-2.17) | 2.25 (1.61-3.15) | <0.001 |
| - Oesophagus (C15) | 337 | 1.47 (1.00-2.17) | 1.00 | 1.18 (0.79-1.75) | 1.01 (0.64-1.58) | 1.28 (0.76-2.14) | 2.74 (1.64-4.57) | <0.001 |
| - Larynx (C32) | 123 | 0.46 (0.26-0.83) | 1.00 | 0.51 (0.29-0.90) | 0.71 (0.40-1.28) | 0.69 (0.34-1.40) | 1.22 (0.60-2.46) | 0.02 |
| Colorectum (C18-20) | 3,878 | 0.99 (0.89-1.09) | 1.00 | 1.02 (0.92-1.14) | 1.23 (1.09-1.39) | 1.32 (1.14-1.54) | 1.53 (1.26-1.85) | <0.001 |
| - Colon (C18) | 2,785 | 1.03 (0.91-1.16) | 1.00 | 1.05 (0.92-1.19) | 1.22 (1.06-1.40) | 1.37 (1.14-1.64) | 1.56 (1.24-1.96) | <0.001 |
| - Rectum (C19-20) | 1,137 | 0.88 (0.73-1.07) | 1.00 | 0.95 (0.78-1.15) | 1.24 (1.01-1.53) | 1.21 (0.92-1.58) | 1.52 (1.11-2.09) | <0.001 |
| Liver (C22) | 380 | 1.16 (0.84-1.61) | 1.00 | 0.83 (0.58-1.19) | 0.84 (0.56-1.26) | 1.32 (0.84-2.07) | 2.71 (1.75-4.19) | <0.001 |
| Breast (C50^b^) | 4,220 | 0.92 (0.84-1.01) | 1.00 | 1.05 (0.96-1.15) | 1.17 (1.05-1.32) | 1.35 (1.11-1.64) | 1.09 (0.68-1.74) | <0.001 |
| **Alcohol-related cancers combined (C00-15;18-20;22;32;50^c^)** | 9,574 | 0.94 (0.88-1.00) | 1.00 | 1.02 (0.95-1.08) | 1.14 (1.05-1.22) | 1.31 (1.19-1.45) | 1.73 (1.52-1.97) | <0.001 |
| **All cancers combined (C00-97;D45-46;47.1;47.3-47.5)** | 31,996 | 0.99 (0.96-1.03) | 1.00 | 1.01 (0.97-1.05) | 1.06 (1.02-1.11) | 1.03 (0.98-1.09) | 1.16 (1.08-1.24) | <0.001 |

Models were adjusted for cancer-specific covariates as listed in Supplementary Table 1. Cancer cases do not sum to totals as some participants were diagnosed with two or more primary cancers. ^a^*p* heterogeneity. ^b^Breast cancer in women only. ICD-10, International Classification of Diseases, version 10.

**Supplementary Table 10. Hazard ratios (HR) and 95% confidence intervals (CI) of cancer risk by pattern of drinking among participants consuming ≥4 drinks per week, with exclusion of the first year of follow-up, in the 45 and Up Study (2005-2019).**

|  |  | **4-7 drinking days per week** | | |  | **1-3 drinking days per week** | | |  |
| --- | --- | --- | --- | --- | --- | --- | --- | --- | --- |
| **Cancer type (ICD-10 code)** | **n cases** | **≥4 to ≤7 drinks** | **>7 to ≤10 drinks** | **>10 drinks** |  | **≥4 to ≤7 drinks** | **>7 to ≤10 drinks** | **>10 drinks** | ***p*_interaction_^a^** |
| Upper aerodigestive tract (C00-15;32) | 730 | 1.00 | 0.98 (0.73-1.30) | 1.24 (0.94-1.64) |  | 0.82 (0.58-1.16) | 1.19 (0.83-1.72) | 1.08 (0.64-1.85) | 0.19 |
| Colorectum (C18-20) | 2,091 | 1.00 | 0.97 (0.83-1.13) | 1.25 (1.08-1.45) |  | 0.99 (0.82-1.19) | 1.13 (0.91-1.41) | 1.16 (0.81-1.65) | 0.36 |
| - Colon (C18) | 1,470 | 1.00 | 0.92 (0.77-1.10) | 1.18 (0.99-1.40) |  | 1.02 (0.83-1.26) | 1.15 (0.89-1.49) | 1.41 (0.94-2.10) | 0.44 |
| - Rectum (C19-20) | 645 | 1.00 | 1.11 (0.82-1.50) | 1.46 (1.09-1.96) |  | 0.95 (0.66-1.35) | 1.14 (0.76-1.72) | 0.70 (0.33-1.48) | 0.15 |
| Breast (C50^b^) | 1,914 | 1.00 | 0.97 (0.85-1.11) | 1.13 (0.98-1.30) |  | 0.94 (0.80-1.10) | 1.04 (0.83-1.31) | 1.70 (1.06-2.71) | 0.13 |
| **Alcohol-related cancers combined (C00-15;18-20;22;32;50^b^)** | 4,870 | 1.00 | 0.96 (0.87-1.06) | 1.29 (1.03-1.63) |  | 0.94 (0.84-1.06) | 1.08 (0.93-1.24) | 1.29 (1.03-1.63) | 0.11 |
| **All cancers combined (C00-97;D45-46;47.1;47.3-47.5)** | 17,424 | 1.00 | 1.01 (0.96-1.07) | 1.12 (1.06-1.18) |  | 1.07 (1.00-1.13) | 1.14 (1.06-1.23) | 1.12 (0.99-1.26) | 0.17 |

Models were adjusted for cancer-specific covariates as listed in Supplementary Table 1. Cancer cases do not sum to totals as some participants were diagnosed with two or more primary cancers. ^a^Test of interaction between days per week and drinks per week. ^b^Breast cancer in women only. ICD-10, International Classification of Diseases, version 10.

**Supplementary Table 11. Hazard ratios (HR) and 95% confidence intervals (CI) of cancer risk by alcohol consumption for additional cancer types in the 45 and Up Study (2005-2019).**

|  |  |  | **HR drinks per week (95% CI)** | | | | | |  | **n cases** | **HR per 10 drink** |  |
| --- | --- | --- | --- | --- | --- | --- | --- | --- | --- | --- | --- | --- |
| **Cancer type (ICD-10 code)** | **n cases** | **Age- and sex-standardised rate^a^** | **0 to <1** | **≥1 to ≤3.5** | **>3.5 to ≤10** | **>10 to ≤20** | **>20 to ≤30** | **>30** | ***p*^b^** | **among drinkers** | **increase per week (95% CI)** | ***p*_trend_^c^** |
| Stomach (C16) | 573 | 25.0 | 1.19 (0.91-1.55) | 1.00 | 1.10 (0.84-1.45) | 0.96 (0.70-1.32) | 0.82 (0.53-1.25) | 0.66 (0.36-1.20) | 0.16 | 368 | 0.88 (0.76-1.02) | 0.08 |
| Pancreas (C25) | 940 | 41.2 | 1.02 (0.83-1.25) | 1.00 | 1.00 (0.81-1.23) | 1.06 (0.83-1.35) | 1.02 (0.74-1.41) | 1.23 (0.82-1.85) | 0.93 | 612 | 1.05 (0.94-1.17) | 0.39 |
| Lung (C33-34) | 2,627 | 108.4 | 1.15 (1.01-1.32) | 1.00 | 1.08 (0.94-1.24) | 1.22 (1.05-1.42) | 1.23 (1.03-1.47) | 1.38 (1.12-1.70) | 0.01 | 1,780 | 1.10 (1.04-1.17) | 0.001 |
| Melanoma (C43) | 4,844 | 197.1 | 0.87 (0.79-0.96) | 1.00 | 1.04 (0.95-1.13) | 1.07 (0.97-1.19) | 1.01 (0.89-1.16) | 1.16 (0.97-1.38) | <0.001 | 3,584 | 1.04 (0.99-1.09) | 0.11 |

Models were adjusted for cancer-specific covariates as listed in Supplementary Table 1. Cancer cases do not sum to totals as some participants were diagnosed with two or more primary cancers. ^a^Standardised rate per 100,000 person-years, standardised by age and sex to the 2006 New South Wales population aged ≥45 years. ^b^*p* heterogeneity. ^c^Linear trend in categories calculated among drinkers only, where participants within each category of alcohol consumption at baseline were assigned the mean level of alcohol consumption they reported at first wave follow-up (median 5.3 years after baseline). ICD-10, International Classification of Diseases, version 10.

**Supplementary Table 12. Hazard ratios (HR) and 95% confidence intervals (CI) of cancer risk by pattern of drinking among participants consuming ≥4 drinks per week for additional cancer types in the 45 and Up Study (2005-2019).**

|  |  | **4-7 drinking days per week** | | |  | **1-3 drinking days per week** | | |  |
| --- | --- | --- | --- | --- | --- | --- | --- | --- | --- |
| **Cancer type (ICD-10 code)** | **n cases** | **≥4 to ≤7 drinks** | **>7 to ≤10 drinks** | **>10 drinks** |  | **≥4 to ≤7 drinks** | **>7 to ≤10 drinks** | **>10 drinks** | ***p*_interaction_^a^** |
| Lung (C33-34) | 1,470 | 1.00 | 1.02 (0.84-1.25) | 1.20 (1.03-1.40) |  | 1.10 (0.90-1.34) | 1.17 (0.89-1.55) | 1.08 (0.78-1.49) | 0.42 |
| Melanoma (C43) | 2,847 | 1.00 | 1.11 (0.97-1.26) | 1.16 (1.04-1.29) |  | 1.19 (1.04-1.35) | 1.27 (1.05-1.54) | 1.03 (0.80-1.33) | 0.12 |

Models were adjusted for cancer-specific covariates as listed in Supplementary Table 1. Cancer cases do not sum to totals as some participants were diagnosed with two or more primary cancers. ^a^Test of interaction between days per week and drinks per week. ICD-10, International Classification of Diseases, version 10.

**Supplementary Table 13. Interaction tests and stratifications for hazard ratios (HR) and 95% confidence intervals (CI) of cancer risk per ten drink increase in weekly alcohol consumption among drinkers for additional cancer types in the 45 and Up Study (2005-2019).**

| **Cancer type (ICD-10 code)** | **n cases** | **Main analysis** | ***p*_interaction (sex)_** | **Never smoked** | **Formerly smoked** | **Currently smoke** | ***p*_interaction (smoking)_** | ***p*_interaction (COB)_** | ***p*_interaction (SES)_** |
| --- | --- | --- | --- | --- | --- | --- | --- | --- | --- |
| Stomach (C16) | 368 | 0.88 (0.76-1.02) | 0.34 | 0.73 (0.53-1.01) | 0.84 (0.69-1.02) | 1.20 (0.86-1.68) | 0.02 | 0.91 | 0.55 |
| Pancreas (C25) | 612 | 1.05 (0.94-1.17) | 0.51 | - | - | - | 0.45 | 0.33 | 0.15 |
| Lung (C33-34) | 1,780 | 1.10 (1.04-1.17) | 0.62 | - | - | - | 0.88 | 0.93 | 0.69 |
| Melanoma (C43) | 3,584 | 1.04 (0.99-1.09) | 0.63 | - | - | - | 0.43 | 0.36 | 0.96 |

Linear trend in categories calculated among drinkers only, where participants within each category of alcohol consumption at baseline were assigned the mean level of alcohol consumption they reported at first wave follow-up (median 5.3 years after baseline). Models were adjusted for cancer-specific covariates as listed in Supplementary Table 1. Cancer cases do not sum to totals as some participants were diagnosed with two or more primary cancers. COB, Country Of Birth. ICD-10, International Classification of Diseases, version 10. SES, Socio-Economic Status.

**Supplementary Table 14. Cumulative absolute risk (%) of cancer diagnosis from age 25 to 85 years in Australia in 2024 by sex and level of alcohol consumption for additional cancer types using hazard ratios from the 45 and Up Study (2005-2019).**

| **Sex and age** | **Pancreas**  **(drinks/week)** | | |  | **Lung**  **(drinks/week)** | | |  | **Melanoma**  **(drinks/week)** | | |
| --- | --- | --- | --- | --- | --- | --- | --- | --- | --- | --- | --- |
|  | **0 to <1** | **≥1 to ≤10** | **>10** |  | **0 to <1** | **≥1 to ≤10** | **>10** |  | **0 to <1** | **≥1 to ≤10** | **>10** |
| **Men** |  |  |  |  |  |  |  |  |  |  |  |
| 25 years | 0.00 | 0.00 | 0.00 |  | 0.00 | 0.00 | 0.00 |  | 0.00 | 0.00 | 0.00 |
| 30 years | 0.00 | 0.00 | 0.00 |  | 0.00 | 0.00 | 0.00 |  | 0.03 | 0.04 | 0.04 |
| 35 years | 0.01 | 0.01 | 0.01 |  | 0.01 | 0.01 | 0.01 |  | 0.10 | 0.11 | 0.11 |
| 40 years | 0.01 | 0.01 | 0.01 |  | 0.02 | 0.03 | 0.03 |  | 0.22 | 0.23 | 0.24 |
| 45 years | 0.03 | 0.03 | 0.04 |  | 0.06 | 0.06 | 0.07 |  | 0.41 | 0.42 | 0.45 |
| 50 years | 0.07 | 0.07 | 0.08 |  | 0.11 | 0.12 | 0.14 |  | 0.74 | 0.76 | 0.80 |
| 55 years | 0.14 | 0.14 | 0.15 |  | 0.26 | 0.27 | 0.31 |  | 1.17 | 1.20 | 1.27 |
| 60 years | 0.25 | 0.26 | 0.27 |  | 0.56 | 0.59 | 0.68 |  | 1.76 | 1.79 | 1.90 |
| 65 years | 0.42 | 0.43 | 0.46 |  | 1.10 | 1.16 | 1.34 |  | 2.50 | 2.55 | 2.70 |
| 70 years | 0.65 | 0.67 | 0.72 |  | 1.84 | 1.93 | 2.23 |  | 3.57 | 3.64 | 3.85 |
| 75 years | 0.98 | 1.00 | 1.08 |  | 2.97 | 3.11 | 3.59 |  | 5.01 | 5.10 | 5.40 |
| 80 years | 1.42 | 1.46 | 1.57 |  | 4.46 | 4.68 | 5.40 |  | 6.70 | 6.83 | 7.23 |
| 85 years | 1.91 | 1.96 | 2.10 |  | 6.07 | 6.37 | 7.34 |  | 8.56 | 8.72 | 9.23 |
| **Women** |  |  |  |  |  |  |  |  |  |  |  |
| 25 years | 0.00 | 0.00 | 0.00 |  | 0.00 | 0.00 | 0.00 |  | 0.00 | 0.00 | 0.00 |
| 30 years | 0.00 | 0.00 | 0.00 |  | 0.00 | 0.01 | 0.01 |  | 0.05 | 0.05 | 0.06 |
| 35 years | 0.01 | 0.01 | 0.01 |  | 0.01 | 0.01 | 0.01 |  | 0.18 | 0.18 | 0.18 |
| 40 years | 0.02 | 0.02 | 0.03 |  | 0.02 | 0.02 | 0.03 |  | 0.33 | 0.33 | 0.34 |
| 45 years | 0.04 | 0.04 | 0.04 |  | 0.05 | 0.06 | 0.06 |  | 0.55 | 0.56 | 0.58 |
| 50 years | 0.07 | 0.07 | 0.07 |  | 0.11 | 0.12 | 0.13 |  | 0.84 | 0.86 | 0.89 |
| 55 years | 0.11 | 0.12 | 0.12 |  | 0.25 | 0.26 | 0.28 |  | 1.18 | 1.21 | 1.25 |
| 60 years | 0.19 | 0.19 | 0.20 |  | 0.58 | 0.60 | 0.66 |  | 1.60 | 1.63 | 1.69 |
| 65 years | 0.30 | 0.30 | 0.32 |  | 1.10 | 1.15 | 1.26 |  | 2.13 | 2.17 | 2.24 |
| 70 years | 0.49 | 0.50 | 0.53 |  | 1.86 | 1.95 | 2.12 |  | 2.77 | 2.83 | 2.92 |
| 75 years | 0.76 | 0.78 | 0.82 |  | 2.94 | 3.08 | 3.35 |  | 3.58 | 3.65 | 3.78 |
| 80 years | 1.10 | 1.13 | 1.18 |  | 4.30 | 4.51 | 4.91 |  | 4.51 | 4.60 | 4.76 |
| 85 years | 1.55 | 1.59 | 1.66 |  | 5.52 | 5.79 | 6.31 |  | 5.37 | 5.47 | 5.66 |

Calculations based on the results of the continuous variable analysis among 45 and Up Study participants consuming ≥1 drink per week. Three categories of drinking were used: 0 to <1 drink per week (never-drinkers, former drinkers and occasional drinkers who consumed <1 drink per week), ≥1 to ≤10 drinks per week (median 5 drinks in men and women), and >10 drinks per week (median 20 drinks in men and 14 drinks in women). As the hazard ratio point estimate for stomach cancer was less than 1, it was not possible to calculate estimates of cumulative absolute risk for stomach cancer.

**Supplementary Table 15. Population attributable fractions for cancer caused by alcohol consumption in the Australian population in 2024 for additional cancer types, derived using risk estimates from the 45 and Up Study (2005-2019) for current drinking and international data for former drinking.**

|  | **Men** |  |  | **Women** |  |  | **Persons** |  |
| --- | --- | --- | --- | --- | --- | --- | --- | --- |
| **Cancer type (ICD-10 code)** | **n alcohol-attributable cases/n total cases** | **PAF (%)** |  | **n alcohol-attributable cases/n total cases** | **PAF (%)** |  | **n alcohol-attributable cases/n total cases** | **PAF (%)** |
| ***Cancer cases attributable to current alcohol consumption*** |  |  |  |  |  |  |  |  |
| Stomach (C16) | -^a^/1,662 | -^a^ |  | -^a^/922 | -^a^ |  | -^a^/2,584 | -^a^ |
| Pancreas (C25) | 133/2,414 | 5.5 |  | 49/2,227 | 2.2 |  | 182/4,641 | 3.9 |
| Lung (C33-34) | 852/7,718 | 11.0 |  | 351/7,404 | 4.7 |  | 1,203/15,122 | 8.0 |
| Melanoma (C43) | 479/11,034 | 4.3 |  | 142/7,930 | 1.8 |  | 621/18,964 | 3.3 |
| **Alcohol-related cancers combined + additional four cancer types**  **(C00-16;18-20;22;25;32-34;43;50^b^)** | 5,540/46,804 | 11.8 |  | 3,593/56,845 | 6.3 |  | 9,133/103,649 | 8.8 |
| **All cancers combined (C00-97;D45-46;47.1;47.3-47.5)** | 5,540/93,504 | 5.9 |  | 3,593/75,974 | 4.7 |  | 9,133/169,478 | 5.4 |
|  |  |  |  |  |  |  |  |  |
| ***Cancer cases attributable to former alcohol consumption*** |  |  |  |  |  |  |  |  |
| Stomach (C16) | 28/1,662 | 1.7 |  | 38/922 | 4.1 |  | 66/2,584 | 2.6 |
| Pancreas (C25) | 37/2,414 | 1.5 |  | 93/2,227 | 4.2 |  | 130/4,641 | 2.8 |
| Lung (C33-34) | -^c^/7,718 | -^c^ |  | -^c^/7,404 | -^c^ |  | -^c^/15,122 | -^c^ |
| Melanoma (C43) | -^c^/11,034 | -^c^ |  | -^c^/7,930 | -^c^ |  | -^c^/18,964 | -^c^ |
| **Alcohol-related cancers combined + additional four cancer types**  **(C00-16;18-20;22;25;32-34;43;50^b^)** | 645/46,804 | 1.4 |  | 227/56,845 | 0.4 |  | 873/103,649 | 0.8 |
| **All cancers combined (C00-97;D45-46;47.1;47.3-47.5)** | 645/93,504 | 0.7 |  | 227/75,974 | 0.3 |  | 873/169,478 | 0.5 |
|  |  |  |  |  |  |  |  |  |
| ***Total cancer cases attributable to alcohol consumption*** |  |  |  |  |  |  |  |  |
| Stomach (C16) | 28/1,662 | 1.7 |  | 38/922 | 4.1 |  | 66/2,584 | 2.6 |
| Pancreas (C25) | 170/2,414 | 7.0 |  | 142/2,227 | 6.4 |  | 312/4,641 | 6.7 |
| Lung (C33-34) | 852/7,718 | 11.0 |  | 351/7,404 | 4.7 |  | 1,203/15,122 | 8.0 |
| Melanoma (C43) | 479/11,034 | 4.3 |  | 142/7,930 | 1.8 |  | 621/18,964 | 3.3 |
| **Alcohol-related cancers combined + additional four cancer types**  **(C00-16;18-20;22;25;32-34;43;50^b^)** | 6,185/46,804 | 13.2 |  | 3,820/56,845 | 6.7 |  | 10,005/103,649 | 9.7 |
| **All cancers combined (C00-97;D45-46;47.1;47.3-47.5)** | 6,185/93,504 | 6.6 |  | 3,820/75,974 | 5.0 |  | 10,005/169,478 | 5.9 |

Hazard ratios used in calculations for the 45 and Up Study were derived from total alcohol consumption as a log-linear variable among participants consuming ≥1 drink per week. For each age group used in the calculation, cancer incidence was attributed to alcohol consumption approximately 10 years earlier in the 2011-2012 Australian Health Survey. ‘n excess cases’ refers to cancer cases attributable to alcohol consumption out of the total number of cases, ‘n cases’. Cancer cases may not sum to totals due to rounding. ^a^As the hazard ratio point estimate for stomach cancer was less than 1, it was not possible to calculate estimates for population attributable fractions for current alcohol consumption for stomach cancer. ^b^Breast cancer in women only. ^c^As relative risks for former alcohol consumption for lung cancer and melanoma were not reported in Rumgay et al., (2021)(9), it was not possible to calculate estimates for population attributable fractions for former alcohol consumption for lung cancer and melanoma. ICD-10, International Classification of Diseases, version 10. PAF, Population Attributable Fraction.

**Supplementary Table 16. Hazard ratios (HR) and 95% confidence intervals (CI) of cancer risk by alcohol consumption, with minimal adjustment for covariates in models, in the 45 and Up Study (2005-2019).**

|  |  | **HR drinks per week (95% CI)** | | | | | |  |
| --- | --- | --- | --- | --- | --- | --- | --- | --- |
| **Cancer type (ICD-10 code)** | **n cases** | **0 to <1** | **≥1 to ≤3.5** | **>3.5 to ≤10** | **>10 to ≤20** | **>20 to ≤30** | **>30** | ***p*^a^** |
| Upper aerodigestive tract (C00-15;32) | 1,324 | 1.00 (0.83-1.20) | 1.00 | 1.01 (0.84-1.21) | 1.16 (0.95-1.42) | 1.70 (1.35-2.15) | 3.20 (2.51-4.08) | <0.001 |
| - Mouth and pharynx (C00-14) | 825 | 0.90 (0.71-1.13) | 1.00 | 1.02 (0.81-1.28) | 1.18 (0.91-1.51) | 1.86 (1.40-2.47) | 2.89 (2.11-3.96) | <0.001 |
| - Oesophagus (C15) | 370 | 1.51 (1.04-2.17) | 1.00 | 1.23 (0.85-1.79) | 1.23 (0.81-1.87) | 1.56 (0.95-2.55) | 3.94 (2.44-6.34) | <0.001 |
| - Larynx (C32) | 136 | 0.65 (0.37-1.14) | 1.00 | 0.60 (0.34-1.04) | 0.95 (0.54-1.67) | 1.16 (0.59-2.29) | 3.18 (1.69-5.99) | <0.001 |
| Colorectum (C18-20) | 4,261 | 1.03 (0.94-1.14) | 1.00 | 1.00 (0.90-1.10) | 1.23 (1.10-1.37) | 1.39 (1.21-1.60) | 1.73 (1.45-2.06) | <0.001 |
| - Colon (C18) | 3,040 | 1.07 (0.95-1.20) | 1.00 | 1.02 (0.90-1.15) | 1.21 (1.06-1.39) | 1.44 (1.21-1.70) | 1.80 (1.46-2.23) | <0.001 |
| - Rectum (C19-20) | 1,271 | 0.92 (0.77-1.10) | 1.00 | 0.91 (0.76-1.09) | 1.22 (1.01-1.48) | 1.30 (1.02-1.67) | 1.63 (1.21-2.20) | <0.001 |
| Liver (C22) | 412 | 1.48 (1.08-2.04) | 1.00 | 0.80 (0.56-1.13) | 0.83 (0.56-1.23) | 1.41 (0.91-2.18) | 3.61 (2.38-5.48) | <0.001 |
| Breast (C50^b^) | 4,609 | 0.92 (0.85-1.00) | 1.00 | 1.07 (0.98-1.17) | 1.21 (1.09-1.35) | 1.33 (1.10-1.60) | 1.11 (0.71-1.74) | <0.001 |
| **Alcohol-related cancers combined (C00-15;18-20;22;32;50^b^)** | 10,471 | 0.97 (0.91-1.03) | 1.00 | 1.02 (0.96-1.09) | 1.18 (1.10-1.27) | 1.42 (1.29-1.56) | 1.99 (1.77-2.25) | <0.001 |
| **All cancers combined (C00-97;D45-46;47.1;47.3-47.5)** | 34,860 | 1.00 (0.97-1.03) | 1.00 | 1.02 (0.98-1.05) | 1.11 (1.07-1.16) | 1.13 (1.07-1.18) | 1.31 (1.23-1.40) | <0.001 |

Models were adjusted for sex. Cancer cases do not sum to totals as some participants were diagnosed with two or more primary cancers. ^a^*p* heterogeneity. ^b^Breast cancer in women only. ICD-10, International Classification of Diseases, version 10.

**Supplementary Table 17. Hazard ratios (HR) and 95% confidence intervals (CI) of cancer risk per ten drink increase in weekly alcohol consumption among drinkers, with different levels of adjustment for covariates, in the 45 and Up Study (2005-2019).**

| **Cancer type (ICD-10 code)** | **n cases** | **Main analysis^a^** | **Model not adjusted for body mass index** | **Minimally adjusted model^b^** |
| --- | --- | --- | --- | --- |
| Upper aerodigestive tract (C00-15;32) | 972 | 1.27 (1.18-1.37) | 1.27 (1.18-1.37) | 1.45 (1.35-1.55) |
| - Mouth and pharynx (C00-14) | 621 | 1.27 (1.16-1.39) | 1.27 (1.16-1.40) | 1.42 (1.29-1.55) |
| - Oesophagus (C15) | 248 | 1.29 (1.11-1.49) | 1.29 (1.11-1.49) | 1.47 (1.27-1.69) |
| - Larynx (C32) | 108 | 1.22 (0.99-1.50) | 1.23 (1.00-1.51) | 1.55 (1.27-1.89) |
| Colorectum (C18-20) | 2,889 | 1.16 (1.11-1.22) | 1.16 (1.11-1.22) | 1.21 (1.16-1.27) |
| - Colon (C18) | 2,012 | 1.18 (1.11-1.25) | 1.18 (1.11-1.25) | 1.22 (1.15-1.29) |
| - Rectum (C19-20) | 914 | 1.15 (1.06-1.25) | 1.15 (1.06-1.25) | 1.21 (1.12-1.32) |
| Liver (C22) | 258 | 1.46 (1.28-1.68) | 1.47 (1.28-1.68) | 1.62 (1.42-1.85) |
| Breast (C50^c^) | 2,891 | 1.18 (1.09-1.28) | 1.17 (1.08-1.27) | 1.17 (1.08-1.26) |
| **Alcohol-related cancers combined (C00-15;18-20;22;32;50^c^)** | 6,923 | 1.19 (1.15-1.23) | 1.19 (1.15-1.23) | 1.25 (1.21-1.29) |
| **All cancers combined (C00-97;D45-46;47.1;47.3-47.5)** | 24,133 | 1.05 (1.03-1.07) | 1.05 (1.03-1.06) | 1.09 (1.07-1.11) |

Linear trend in categories calculated among drinkers only, where participants within each category of alcohol consumption at baseline were assigned the mean level of alcohol consumption they reported at first wave follow-up (median 5.3 years after baseline). Cancer cases do not sum to totals as some participants were diagnosed with two or more primary cancers. ^a^Models were adjusted for cancer-specific covariates as listed in Supplementary Table 1. ^b^Models were adjusted for sex. ^c^Breast cancer in women only. ICD-10, International Classification of Diseases, version 10.

**Supplementary Table 18. Hazard ratios (HR) and 95% confidence intervals (CI) of cancer risk by pattern of drinking among participants consuming ≥4 drinks per week, with minimal adjustment for covariates in models, in the 45 and Up Study (2005-2019).**

|  |  | **4-7 drinking days per week** | | |  | **1-3 drinking days per week** | | |  |
| --- | --- | --- | --- | --- | --- | --- | --- | --- | --- |
| **Cancer type (ICD-10 code)** | **n cases** | **≥4 to ≤7 drinks** | **>7 to ≤10 drinks** | **>10 drinks** |  | **≥4 to ≤7 drinks** | **>7 to ≤10 drinks** | **>10 drinks** | ***p*_interaction_^a^** |
| Upper aerodigestive tract (C00-15;32) | 790 | 1.00 | 1.25 (0.96-1.63) | 1.69 (1.37-2.09) |  | 0.98 (0.73-1.31) | 1.76 (1.24-2.50) | 1.76 (1.17-2.64) | 0.27 |
| Colorectum (C18-20) | 2,282 | 1.00 | 1.03 (0.89-1.19) | 1.38 (1.24-1.55) |  | 1.06 (0.91-1.24) | 1.31 (1.05-1.64) | 1.47 (1.13-1.91) | 0.41 |
| - Colon (C18) | 1,591 | 1.00 | 1.01 (0.85-1.20) | 1.36 (1.19-1.55) |  | 1.10 (0.92-1.32) | 1.36 (1.04-1.78) | 1.69 (1.24-2.30) | 0.46 |
| - Rectum (C19-20) | 719 | 1.00 | 1.10 (0.84-1.44) | 1.51 (1.22-1.86) |  | 1.02 (0.77-1.35) | 1.27 (0.85-1.88) | 1.15 (0.70-1.88) | 0.40 |
| Breast (C50^b^) | 2,100 | 1.00 | 1.09 (0.95-1.24) | 1.19 (1.06-1.33) |  | 1.00 (0.87-1.14) | 1.17 (0.93-1.49) | 1.28 (0.89-1.84) | 0.81 |
| **Alcohol-related cancers combined (C00-15;18-20;22;32;50^b^)** | 5,307 | 1.00 | 1.11 (1.01-1.22) | 1.37 (1.27-1.47) |  | 1.05 (0.96-1.16) | 1.32 (1.14-1.53) | 1.46 (1.22-1.75) | 0.37 |
| **All cancers combined (C00-97;D45-46;47.1;47.3-47.5)** | 18,929 | 1.00 | 1.08 (1.03-1.14) | 1.20 (1.16-1.25) |  | 1.12 (1.07-1.18) | 1.25 (1.16-1.34) | 1.24 (1.13-1.35) | 0.11 |

Models were adjusted for sex. Cancer cases do not sum to totals as some participants were diagnosed with two or more primary cancers. ^a^Test of interaction between days per week and drinks per week. ^b^Breast cancer in women only. ICD-10, International Classification of Diseases, version 10.

**Supplementary Table 19. Hazard ratios (HR) and 95% confidence intervals (CI) of cancer risk by alcohol consumption, without adjustment for body mass index, in the 45 and Up Study (2005-2019).**

|  |  | **HR drinks per week (95% CI)** | | | | | |  |
| --- | --- | --- | --- | --- | --- | --- | --- | --- |
| **Cancer type (ICD-10 code)** | **n cases** | **0 to <1** | **≥1 to ≤3.5** | **>3.5 to ≤10** | **>10 to ≤20** | **>20 to ≤30** | **>30** | ***p*^a^** |
| Upper aerodigestive tract (C00-15;32) | 1,324 | 0.93 (0.77-1.11) | 1.00 | 0.99 (0.82-1.19) | 1.06 (0.87-1.30) | 1.39 (1.10-1.76) | 2.21 (1.72-2.84) | <0.001 |
| - Mouth and pharynx (C00-14) | 825 | 0.82 (0.65-1.04) | 1.00 | 1.02 (0.81-1.28) | 1.14 (0.88-1.47) | 1.63 (1.22-2.17) | 2.13 (1.54-2.96) | <0.001 |
| - Oesophagus (C15) | 370 | 1.45 (1.00-2.09) | 1.00 | 1.17 (0.80-1.70) | 1.05 (0.69-1.61) | 1.20 (0.73-1.97) | 2.59 (1.59-4.23) | <0.001 |
| - Larynx (C32) | 136 | 0.57 (0.33-1.01) | 1.00 | 0.56 (0.32-0.98) | 0.76 (0.43-1.35) | 0.78 (0.39-1.54) | 1.65 (0.86-3.18) | 0.007 |
| Colorectum (C18-20) | 4,261 | 0.98 (0.89-1.08) | 1.00 | 1.00 (0.90-1.10) | 1.20 (1.07-1.34) | 1.30 (1.12-1.50) | 1.49 (1.24-1.78) | <0.001 |
| - Colon (C18) | 3,040 | 1.02 (0.91-1.15) | 1.00 | 1.02 (0.90-1.15) | 1.19 (1.04-1.36) | 1.35 (1.13-1.60) | 1.58 (1.27-1.97) | <0.001 |
| - Rectum (C19-20) | 1,271 | 0.87 (0.72-1.04) | 1.00 | 0.92 (0.77-1.10) | 1.20 (0.99-1.47) | 1.21 (0.94-1.55) | 1.36 (1.00-1.84) | <0.001 |
| Liver (C22) | 412 | 1.32 (0.96-1.82) | 1.00 | 0.85 (0.60-1.21) | 0.87 (0.58-1.29) | 1.34 (0.86-2.08) | 2.89 (1.88-4.44) | <0.001 |
| Breast (C50^b^) | 4,609 | 0.95 (0.87-1.04) | 1.00 | 1.05 (0.96-1.15) | 1.18 (1.06-1.32) | 1.31 (1.08-1.58) | 1.12 (0.71-1.74) | <0.001 |
| **Alcohol-related cancers combined (C00-15;18-20;22;32;50^b^)** | 10,471 | 0.96 (0.91-1.02) | 1.00 | 1.01 (0.95-1.07) | 1.13 (1.05-1.21) | 1.30 (1.18-1.43) | 1.72 (1.52-1.95) | <0.001 |
| **All cancers combined (C00-97;D45-46;47.1;47.3-47.5)** | 34,860 | 0.99 (0.96-1.03) | 1.00 | 1.00 (0.96-1.03) | 1.06 (1.01-1.10) | 1.03 (0.97-1.08) | 1.15 (1.08-1.23) | <0.001 |

Models were adjusted for cancer-specific covariates as listed in Supplementary Table 1, except for body mass index. Cancer cases do not sum to totals as some participants were diagnosed with two or more primary cancers. ^a^*p* heterogeneity. ^b^Breast cancer in women only. ICD-10, International Classification of Diseases, version 10.

**Supplementary Table 20. Hazard ratios (HR) and 95% confidence intervals (CI) of cancer risk by pattern of drinking among participants consuming ≥4 drinks per week, without adjustment for body mass index, in the 45 and Up Study (2005-2019).**

|  |  | **4-7 drinking days per week** | | |  | **1-3 drinking days per week** | | |  |
| --- | --- | --- | --- | --- | --- | --- | --- | --- | --- |
| **Cancer type (ICD-10 code)** | **n cases** | **≥4 to ≤7 drinks** | **>7 to ≤10 drinks** | **>10 drinks** |  | **≥4 to ≤7 drinks** | **>7 to ≤10 drinks** | **>10 drinks** | ***p*_interaction_^a^** |
| Upper aerodigestive tract (C00-15;32) | 790 | 1.00 | 1.20 (0.92-1.58) | 1.36 (1.10-1.69) |  | 0.90 (0.67-1.20) | 1.42 (1.00-2.02) | 1.19 (0.79-1.81) | 0.41 |
| Colorectum (C18-20) | 2,282 | 1.00 | 1.02 (0.88-1.19) | 1.32 (1.17-1.48) |  | 1.03 (0.88-1.20) | 1.23 (0.98-1.55) | 1.31 (1.00-1.70) | 0.45 |
| - Colon (C18) | 1,591 | 1.00 | 1.01 (0.84-1.20) | 1.30 (1.14-1.49) |  | 1.07 (0.90-1.28) | 1.29 (0.98-1.70) | 1.54 (1.12-2.10) | 0.55 |
| - Rectum (C19-20) | 719 | 1.00 | 1.09 (0.83-1.43) | 1.40 (1.13-1.74) |  | 0.97 (0.73-1.29) | 1.16 (0.78-1.72) | 0.97 (0.59-1.59) | 0.36 |
| Breast (C50^b^) | 2,100 | 1.00 | 1.08 (0.95-1.23) | 1.19 (1.06-1.33) |  | 1.00 (0.88-1.15) | 1.20 (0.95-1.53) | 1.35 (0.94-1.95) | 0.66 |
| **Alcohol-related cancers combined (C00-15;18-20;22;32;50^b^)** | 5,307 | 1.00 | 1.08 (0.99-1.19) | 1.26 (1.17-1.36) |  | 1.01 (0.92-1.11) | 1.23 (1.06-1.42) | 1.29 (1.08-1.55) | 0.43 |
| **All cancers combined (C00-97;D45-46;47.1;47.3-47.5)** | 18,929 | 1.00 | 1.06 (1.01-1.12) | 1.14 (1.09-1.18) |  | 1.10 (1.04-1.16) | 1.19 (1.10-1.28) | 1.15 (1.05-1.26) | 0.20 |

Models were adjusted for cancer-specific covariates as listed in Supplementary Table 1, except for body mass index. Cancer cases do not sum to totals as some participants were diagnosed with two or more primary cancers. ^a^Test of interaction between days per week and drinks per week. ^b^Breast cancer in women only. ICD-10, International Classification of Diseases, version 10.

**Supplementary Table 21. Violations of the proportional hazards assumption in models.**

| **Cancer type (ICD-10 code)** | ***p*_violation_** | **Variables in violation** |
| --- | --- | --- |
| Upper aerodigestive tract (C00-15;32) | 0.19 | - |
| - Mouth and pharynx (C00-14) | 0.68 | - |
| - Oesophagus (C15) | 0.56 | - |
| - Larynx (C32) | 0.82 | - |
| Colorectum (C18-20) | 0.14 | - |
| - Colon (C18) | 0.003 | Alcohol consumption, education, partner status^a^, smoking status and intensity, BMI, fibre intake, MHT use |
| - Rectum (C19-20) | 0.93 | - |
| Liver (C22) | 0.01 | Partner status^a^, smoking status and intensity, BMI |
| Breast (C50^b^) | 0.03 | Partner status, smoking status and intensity, BMI, menopausal status^a^, HC use, MHT use^a^, breast screening history |
| **Alcohol-related cancers combined (C00-15;18-20;22;32;50^b^)** | <0.001 | Sex^a^, education, partner status^a^, country of birth, smoking status and intensity, breastfeeding duration, menopausal status, HC use, MHT use, breast screening history |
| **All cancers combined (C00-97;D45-46;47.1;47.3-47.5)** | <0.001 | Sex^a^, education, partner status^a^, smoking status and intensity, menopausal status^a^, MHT use, aspirin use^a^, prostate screening history^a^, breast screening history |
| Stomach (C16) | 0.02 | Education, partner status^a^, smoking status and intensity |
| Pancreas (C25) | 0.91 | - |
| Lung (C33-34) | 0.08 | - |
| Melanoma (C43) | <0.001 | Sex^a^, remoteness, education, country of birth^a^, skin tone^a^ |

^a^Plotting the log-log graphs did not reveal clear proportional hazards violations for this variable. ^b^Breast cancer in women only. BMI, Body Mass Index. HC, Hormonal Contraceptive. ICD-10, International Classification of Diseases, version 10. MHT, Menopausal Hormone Therapy.

**Supplementary Table 22. Cox models stratified by age for models with violations of the proportional hazards assumption for alcohol consumption.**

|  |  | **HR drinks per week (95% CI)** | | | | | |  |
| --- | --- | --- | --- | --- | --- | --- | --- | --- |
| **Cancer type (ICD-10 code) and age group** | **n cases** | **0 to <1** | **≥1 to ≤3.5** | **>3.5 to ≤10** | **>10 to ≤20** | **>20 to ≤30** | **>30** | ***p*^a^** |
| **Colon (C18) – Main analysis** | **3,040** | **1.02 (0.91-1.14)** | **1.00** | **1.03 (0.91-1.16)** | **1.20 (1.04-1.37)** | **1.36 (1.14-1.61)** | **1.58 (1.27-1.97)** | **<0.001** |
| < 59 years | 580 | 0.95 (0.74-1.22) | 1.00 | 0.97 (0.76-1.25) | 0.85 (0.62-1.16) | 1.12 (0.76-1.64) | 1.29 (0.81-2.04) | 0.54 |
| ≥ 59 years | 2,460 | 1.04 (0.91-1.19) | 1.00 | 1.04 (0.91-1.20) | 1.30 (1.12-1.51) | 1.43 (1.18-1.74) | 1.68 (1.31-2.15) | <0.001 |

Models were adjusted for cancer-specific covariates as listed in Supplementary Table 1. ^a^*p* heterogeneity. ICD-10, International Classification of Diseases, version 10.

**Supplementary material references**

1. Australian Bureau of Statistics. Alcohol consumption. Canberra: ABS; 2022.

2. Australian Institute of Health and Welfare. Cancer Data in Australia. Canberra: AIHW; 2024.

3. Banks E, Joshy G, Weber MF, Liu B, Grenfell R, Egger S, et al. Tobacco smoking and all-cause mortality in a large Australian cohort study: findings from a mature epidemic with current low smoking prevalence. BMC Med. 2015;13:38.

4. Sarich P, Canfell K, Egger S, Banks E, Joshy G, Grogan P, et al. Alcohol consumption, drinking patterns and cancer incidence in an Australian cohort of 226,162 participants aged 45 years and over. Br J Cancer. 2021;124(2):513-23.

5. Sarich P, Canfell K, Egger S, Banks E, Joshy G, Grogan P, et al. Alcohol consumption, drinking patterns and cause-specific mortality in an Australian cohort of 181,607 participants aged 45 years and over. Public Health. 2025;239:230-41.

6. National Health and Medical Research Council. Australian guidelines to reduce health risks from drinking alcohol. Canberra: NHMRC; 2020 [Available from: <https://www.nhmrc.gov.au/health-advice/alcohol>.

7. Australian Bureau of Statistics. 4364.0.55.001 - Australian Health Survey: First Results, 2011-12. Canberra: ABS; 2012. Contract No.: 4364.0.55.001.

8. Wilson LF, Baade PD, Green AC, Jordan SJ, Kendall BJ, Neale RE, et al. The impact of reducing alcohol consumption in Australia: An estimate of the proportion of potentially avoidable cancers 2013-2037. Int J Cancer. 2019;145(11):2944-53.

9. Rumgay H, Shield K, Charvat H, Ferrari P, Sornpaisarn B, Obot I, et al. Global burden of cancer in 2020 attributable to alcohol consumption: a population-based study. Lancet Oncol. 2021;22(8):1071-80.

10. Holmes J, Meier PS, Booth A, Guo Y, Brennan A. The temporal relationship between per capita alcohol consumption and harm: a systematic review of time lag specifications in aggregate time series analyses. Drug Alcohol Depend. 2012;123(1-3):7-14.

11. Australian Government Department of Health. Make your Move - Sit less - Be active for life! Canberra: Australian Government Department of Health; 2014.
